# Activation of Corticothalamic Layer 6 Cells Decreases Angular Tuning in Mouse Barrel Cortex

**DOI:** 10.3389/fncir.2019.00067

**Published:** 2019-11-01

**Authors:** François Philippe Pauzin, Nadja Schwarz, Patrik Krieger

**Affiliations:** Department of Systems Neuroscience, Faculty of Medicine, Ruhr University Bochum, Bochum, Germany

**Keywords:** layer 6, direction selectivity, excitation inhibition balance, NTSR1, GAD2

## Abstract

In the mouse whisker system, the contribution of L6 corticothalamic cells (L6 CT) to cortical and thalamic processing of the whisker deflection direction was investigated. A genetically defined population of L6 CT cells project to infragranular GABAergic interneurons that hyperpolarize neurons in somatosensory barrel cortex (BC). Optogenetic activation of these neurons switched BC to an adapted mode in which excitatory cells lost their angular tuning. In contrast, however, this was not the case with a general activation of inhibitory interneurons via optogenetic activation of Gad2-expressing cells. The decrease in angular tuning, when L6 CT cells were activated, was due to changes in cortical inhibition, and not inherited from changes in the thalamic output. Furthermore, L6 CT driven cortical inhibition, but not the general activation of GABAergic interneurons, abolished adaptation to whisker responses. In the present study, evidence is presented that a subpopulation of L6 CT activates a specific circuit of GABAergic interneurons that will predispose neocortex toward processing of tactile information requiring multiple whisker touches, such as in a texture discrimination task.

## Introduction

Tactile imagery is described based on our sense of touch. The characteristics of a tactile sensory stimuli is encoded as a difference in neuronal response properties. A feature of some neurons in the whisker-to-barrel pathway (Kleinfeld et al., 2006; Diamond and Arabzadeh, 2013; Petersen, 2014; Krieger and Groh, 2015; Bale and Maravall, 2018; de Kock et al., 2018), is that they respond differently depending on the angular direction in which the whisker is displaced. A property referred to as directional selectivity (Zucker and Welker, 1969; Simons, 1978; Simons and Carvell, 1989; Lichtenstein et al., 1990; Shoykhet et al., 2000; Bruno and Simons, 2002; Minnery and Simons, 2003; Minnery et al., 2003; Timofeeva et al., 2003; Bale and Petersen, 2009). In somatosensory barrel cortex, one mechanism underlying directional selectivity is latency tuning of excitatory inputs (Wilent and Contreras, 2005b). Excitatory responses to the preferred direction, i.e., the direction with the largest response, have shorter latencies compared to those of other directions. The direction-dependent timing difference could involve thalamic and cortical mechanisms. In a proposed computational model (Puccini et al., 2006), the degree of directional selectivity is strongly dependent on the mean deflection frequency; selectivity is weakened at high frequencies (Lee and Simons, 2004). According to this model the fact that whiskers are more sensitive to a deflection in a certain angle, is a property more important for detection of object position, and less so during sustained high frequency whisking, as e.g., during texture discrimination. The importance of directional selectivity for encoding sensory information thus depends on the stimulus context. In the present study optogenetics was used to modulate brain activity in the anesthetized mouse, to investigate how directional selectivity is affected by changes in the excitation/inhibition balance. Cortical activity was decreased either via direct light activation of GABAergic neurons or indirectly via light activation of a subpopulation of layer 6 corticothalamic cells (L6-Ntsr1 CT cells) known to decrease cortical activity. To study the L6 CT circuit, we thus used the GN220 Ntsr1-Cre mouse line where a population of layer 6 CT cells with both cortical and thalamic connections is labeled (Olsen et al., 2012; Mease et al., 2014; Crandall et al., 2017). In the somatosensory system, optogenetics has been used to study these cells *in vitro* (Kim et al., 2014; Crandall et al., 2015) and *in vivo* (Mease et al., 2014; Pauzin and Krieger, 2018). L6-Ntsr1 CT cells located in the upper part of L6 project to the ventral posterior medial nucleus (VPM), whereas the deeper L6-Ntsr1 cell project to VPM and posterior medial nucleus (POm) of the somatosensory thalamus (Zhang and Deschenes, 1997; Chevee et al., 2018). In the somatosensory, visual and auditory systems, electrophysiology recordings show that optogenetic activation of L6-Ntsr1 cells induce a net suppression of spontaneous and sensory-evoked activity in the cortex via direct connections to local fast-spiking (FS) inhibitory GABAergic interneurons that modulates sensory gain in all layers of cortex (Bortone et al., 2014; Kim et al., 2014; Guo et al., 2017). In the present study, the aim was to investigate the effect on angular tuning in somatosensory barrel cortex layers 4 and 5, when there is a reduction in cortical activity either via activation of L6-Ntsr1 CT pyramidal cells or Gad2 expressing GABAergic interneurons. In addition, the aim was to disentangle the relative importance of thalamic and cortical activity to the angular tuning of neurons. We find that the L6-CT cells activate a specific GABAergic microcircuit, and in effect, cause a decrease in angular tuning, and that this is not due to changes inherited from thalamus. The importance of a local inhibitory network for angular tuning relates to the more general question of the organization of excitatory and inhibitory cells into different microcircuits within a column (Krieger et al., 2007; Groh et al., 2010; Defelipe et al., 2012; Markram et al., 2015; Fox, 2018).

## Materials and Methods

All experiments were in accordance with the local government ethics committee (Landesamt für Natur, Umwelt und Verbraucherschutz, Nordrhein-Westfalen). Extracellular recordings in somatosensory cortex and thalamus were performed in 6 Gad2-IRES-cre (Stock number: 010802; Jackson Laboratory) mice (2 males, 4 females) and 14 Ntsr1-cre (GENSAT, founder line GN220) mice (7 males, 7 females).

### Stereotaxic Virus Injections

Stereotaxic injections of male and female Ntsr1-cre mice (median age = 6.8 months) and Gad2-cre (median age = 2.7 months) were done using ketamine (60 mg/kg), xylazine (12 mg/kg) anesthesia with the addition of acepromazine (0.6 mg/kg) as a sedative. The body temperature was kept constant (37°C) using a heating pad (5 × 12.5 cm, 40-90-2-07, FHC) connected to a temperature controller (DC Temperature Controller 40-90-8D, FHC, Bowdoin, ME, United States). Animals were placed in a stereotaxic frame (Model 1900; David Kopf Instruments, Tujunga, CA, United States). After a small incision was made in the skin, a craniotomy was made over barrel cortex at coordinates 3.0/3.1 mm lateral and 1.6/1.7 mm posterior to bregma. Five hundred nl (range: 400–800 nl) of Adeno-associated viral particles [AAV1/2-double floxed-hChR2(H134R)-mCherry-WPRE-polA] encoding for ChR2-mCherry (GeneDetect, New Zealand) were injected at a depth of 0.9 mm under the dura. Mice were sutured and housed in their cages until the experiment was performed 14–20 days after virus injection.

### Experimental Protocol

To immobilize the animal, anesthesia was first induced by isoflurane 5% (vol/vol) in O_2_ via a vaporizer (EZ-7000; E-Z Anesthesia, Palmer, PA, United States) at 1 L/min. For animal surgery and electrophysiology recordings, animals were anesthetized with an intraperitoneal injection of urethane (1–1.5 g/kg animal weight; Sigma-Aldrich, United States) with acepromazine (0.5 mg/kg) dissolved in saline (NaCl 0.9%). When necessary 1–3 more injections of urethane (0.05–0.1 g/kg animal weight) were done during the experiment to ensure that the animal was not spontaneously whisking. To ensure a stable depth of anesthesia, the breathing cycle (350–500 ms from peak to peak) was monitored using a pressure sensitive piezo element (Zehendner et al., 2013). The craniotomy made 2–3 weeks before, during the virus injection, was still visible facilitating the appropriate placement of the recording electrode after re-drilling the skull carefully. The animal’s head was fixed on a metal plate allowing stable and long-time juxtacellular recordings of single units. Cells were filled with biocytin via electroporation at the completion of the recording in order to identify the cell depth relative to pia. All electrophysiology recordings were done in the left hemisphere, and whiskers were deflected on the animal’s right whisker pad. The increase of the pipette resistance, measured in current-clamp, was used to find single-unit spikes. *In vivo* juxtacellular recordings and biocytin fillings were made with 4-6 MΩ patch pipettes pulled from borosilicate filament glass (Hilgenberg GmbH, Germany, OD: 1.5mm: ID: 0.86mm) on a Sutter P-1000 puller (Sutter Instruments, Novato, CA, United States). Pipettes were filled with extracellular solution: (in mM) 135 NaCl, 5.4 KCl, 1.8 CaCl_2_, 1 MgCl_2_, 5 HEPES and 20 mg/ml biocytin (Sigma-Aldrich; United States), pH adjusted to 7.2 with NaOH. The bath solution on top of the animal head was saline (0.9% NaCl). Signals were digitized between 10 and 50 kHz with a DigiData 1300 (Axon Instruments) and were acquired using pClamp 8 software (Axon Instruments). Spike sorting (threshold and template search; pClamp 8) was done to isolate single-units. The stimulation light (470 nm) was delivered by a LED light source (M470F1; Thorlabs, Newton, NJ, United States) while single neurons were recorded. The output power from the LED driver (DC2100, Thorlabs, Newton, NJ, United States) was regulated by voltage output from a pulse stimulator (Master-8, AMPI) and was measured at the fiber (400 μm diameter; 0.39 NA multimode fiber; M82L01, Thorlabs) tip with a power meter (PM100D; Thorlabs, Newton, NJ, United States).

The maximum number of L6-Ntsr1 cells that could be activated by light stimulation is estimated from a total layer 6 cell density of 24020 cells/mm^3^ (Angenstein et al., 2008) and that 65% of the cells were L6-Ntsr1 cells (Kim et al., 2014). This gives a L6-Ntsr1 cell density of approximately 15600 cells/mm^3^ (including both excitatory and inhibitory neurons). The illumination volume was calculated under the basic assumption of geometrical dispersion (Stark et al., 2012) and above-threshold light intensity in the entire illumination volume. The numerical aperture is related to the angle at which light will exit the fiber: NA = n_i_^∗^ sin(θ) where n_i_ is the scattering coefficient of the media (1.36 for the gray matter, Vo-Dinh, 2003; Aravanis et al., 2007). The angle (θ) is calculated as θ = arcsin (NA/n_i_) = arcsin (0.39/1.36) ≈ 16.6°. To calculate the illumination volume in layer 6 the volume was calculated as the difference between one frustum (Volume V_1_) with height (h) 0.95 mm, radius *r* = 0.2 mm (optical fiber radius) and radius R = r + tan (16.6°)^∗^h (0.95 mm was the deepest L6-Ntsr1 cell recorded from; the assumption is thus conservative since L6 continues to 1200 mm), and one volume (V_2_) with height 0.775 mm. The illuminated volume in L6 = V_1_ – V_2_ = 0.3680 – 0.2532 = 0.1148 mm^3^. This gives an upper estimate of ∼1800 (15600 cells/mm^3^^∗^ 0.1148 mm^3^) activated L6-Ntsr1 cells.

Cells (*n* = 3, three animals) classified as L6-Ntsr1 [selection criteria was previously (Pauzin and Krieger, 2018) confirmed with histology] responded with a spike within 8 ± 1 ms (mean/SD) of light onset. Response probability was 100% and light intensity used was <2.5 mW. Three cells in L5 (not included in the analysis of directional selectivity), that increased there spiking with optogenetic stimulation were not considered Ntsr1 cells because they had a lower response probability and longer response latency (depth/response probability/latency: 760 μm/98%/70 ms; 619 μm/44%/4 ms; 710 μm/26%/115 ms). These are thus likely excitatory cells indirectly activated by L6-Ntsr1 projections to L5. Cells (*n* = 4, 2 animals) classified as Gad2-positive responded to every light pulse, and with a latency of 6 ± 1 ms (mean ± SD) from light onset (cell depth: 200, 451, 504, and 547 μm) A note on the type of Gad2-expresing cells that were recruited. The light source for photo-stimulation is applied at the cortical surface, and since there is a depth-dependent efficiency of light transmission there could be a bias for recruiting cells in the upper layers, although no evidence was found for such bias. Furthermore, the expression of the AAV1/2 might not be homogenous among the Gad2-cre expressing interneuron cell types.

### Histology

**FIGURE 1:**
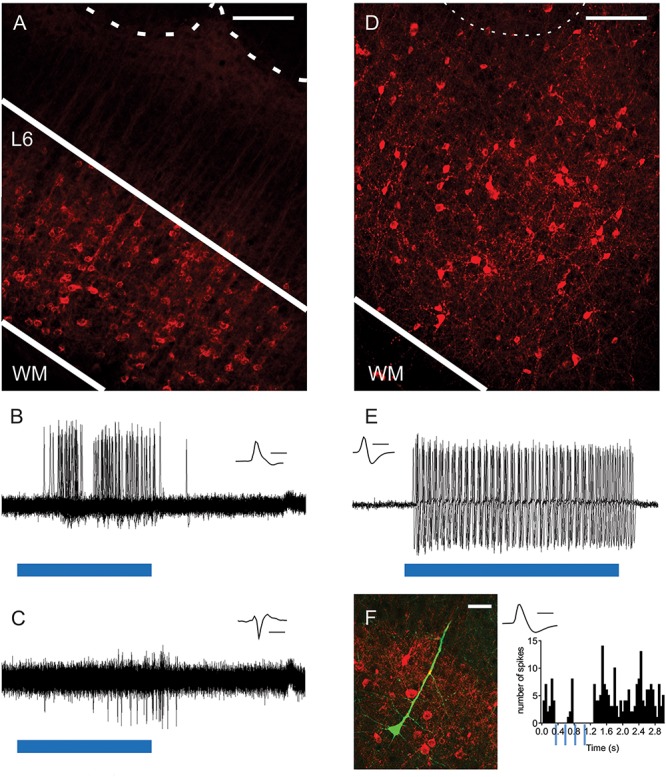
Optogenetic activation of L6-Ntsr1 and Gad2 cells expressing channelrhodopsin. **(A)** ChR2-mCherry expression in L6-Ntsr1 cells in the primary somatosensory barrel cortex. Fluorescent (red) somata in L6 and dendritic tufts and terminals in L5. The dotted lines represent the lower border of layer 4 barrels. **(B)** *In vivo* juxtacellular recording of a L6-Ntsr1 cell expressing channelrhodopsin that responds with spikes to the 50ms light stimulation (blue bar; 0.6 mW). Recordings show the overlay of 50 sweeps. **(C)** *In vivo* juxtacellular recording (overlay of 50 sweeps) of a putative fast-spiking (FS) L6 interneuron (depth: 872 μm). In this example the interneuron was driven via a relatively weak photoactivation of L6-Ntsr1 cells (0.6 mW, blue bar is 50 ms) (Increasing light intensity decreased spike latency, see Supplementary Figure S1). **(D)** Gad2-interneurons expressing ChR2-mCherry (red) were found in all layers in barrel cortex (see also Supplementary Figure S3). **(E)** *In vivo* juxtacellular recording from a L2/3 Gad2-cre expressing interneuron labeled with ChR2-mCherry. The interneuron responded with spikes to light stimulation (0.6 mW; blue bar is 80 ms). Recordings show the overlay of 5 sweeps. **(F)** (*Left*) Image of a labeled pyramidal neuron (green) and ChR2-mCherry expressing Gad2-cre cells (*Right*) PSTH showing that spiking in this pyramidal cell decreased with light stimulation of Gad2 cells (4 light pulses each 80 ms at 4Hz). Scale bars: **(A,D)** = 150 μm; **(F)** = 30 μm. Inset **(B,C,E,F)** Spike shape of the recorded neuron, scale bar = 1 ms. WM, white matter.

Cells (an example shown in Figure 1F) were filled with biocytin during the *in vivo* recording using an electroporation method (Pinault, 1996; Narayanan et al., 2014). After the experiment, the animal was perfused transcardially with 4% paraformaldehyde in 0.1 M phosphate buffer. The brain was removed and post-fixated for at least 24 h in 4% paraformaldehyde. Cell location and morphologies were determined by tissue staining with streptavidin-Alexa-Fluor 488 conjugate (S11223, Thermo Fisher Scientific) or DAB (3,3′-Diaminobenzidine tetrahydrochloridehydrate; D5637, Sigma-Aldrich, United States) as previously described (Groh and Krieger, 2013).

### Determining the Layer Specific Position of Each Cell

Cortical layer position was based on the distance to pia determined by microdrive depth, which in three cells was verified by histological staining [difference in depth estimate was ∼12 μm (data not shown). This corresponds to that found in a larger data set (*n* = 11) using the same recording technique (Pauzin and Krieger, 2018)]. Each cell was assigned to a respective layer based on the recording (microdrive) depth. The depth range used to classify cells was for L2/3: 65 – 319 μm; L4: 320 – 539 μm; L5: 540 – 774 μm; L6: >775 μm. These layer borders correspond to that determined by anatomical techniques (Groh et al., 2010). In this study, we recorded excitatory cells in L4 and L5. The range and median depth of the recorded cells [data from in total 18 animals (6 Gad2 animals and 12 Ntsr1 animals)] were: L4: median depth: 410 μm, range: 333–539 μm (*n* = 25) and L5: median depth: 657 μm, range: 548–763 μm (*n* = 24).

### Separating Excitatory Cells From Interneurons in the *in vivo* Electrophysiology Recordings

Neurons were classified as excitatory cells or inhibitory interneurons based on the shape of their action potentials using a standard classification method (Armstrong-James and Fox, 1987; Bruno and Simons, 2002; Juczewski et al., 2016). Fast-spiking units (presumably interneurons) were distinguished from regular-spiking units (presumably excitatory cells) based on the spike peak-to-trough duration (the interneurons had a very short duration < 0.4 ms), a symmetrical up and down deflection (integral for the interneurons is close to zero). Based on these variables, cells were classified using the K-means clustering (*n* = 2 clusters) method. A principal component analysis shows that PC1 explained 95% of the variance. The “fast” waveform used as the primary criteria to differentiate excitatory from inhibitory cells, might bias the selection of putative GABAergic interneurons to be of the fast-spiking type, rather than regular-waveform GABAergic interneurons.

### Measures of Whisker-Evoked and Spontaneous Spiking

Whisker deflection duration for each pulse was 25 ms with a piezo-deflection amplitude of about 1.0 mm, and with 5 stimulation repeats for each angle per full turn. The whisker was deflected using a ramp-and-hold movement. The peak ramp velocity was estimated to 100 mm/s to the max displacement, which includes displacement due to ringing. The whisker tip (∼10 mm from the base) was put into glass capillary glued to a piezo wafer (PL127.10; PI Ceramics, Germany). The piezo was controlled with a piezo amplifier and filter (Sigmann Elektronik; Germany), and was attached to a motor (Incremental encoder IE3-1024; Faulhaber). The starting whisker deflection angle was chosen randomly. Whiskers were deflected 4 times at 4 Hz repeated every 5 s. After five repeats of the 4 Hz train, the piezo was automatically turned by 45° clockwise. An average of ∼3 full turns per condition was used per each recorded cell (Ntsr1 data: 3.1 turns; Gad2: 3.3 turns). For the control condition, only the piezo was triggered, with no photostimulation. To investigate the effect of L6-Ntsr1 cell activation, the blue light (470 nm) for activation of ChR2 was applied 100 ms before the onset of the first whisker deflection and stopped after the last whisker deflection (the light pulse was thus 875 ms in total). This light stimulation protocol induced a stable level of spiking in the L6-Ntsr1 cells at the time of whisker deflections (Pauzin and Krieger, 2018). To investigate the effect of Gad2-activation, the blue light (470 nm) for activation of ChR2 was applied 10 ms before the onset of each whisker deflection and lasted for 80 ms [thus in total the cell was exposed to a 320 ms (4 pulses ^∗^ 80 ms) light pulse per sweep]. Short 80 ms pulses were chosen to get a stable spiking in the Gad2-GABAergic cells with each whisker deflection. For each cell the principal whisker was defined and used in the experiments. The principal whisker is the whisker that evoked the highest response, as determined by using a handheld probe to touch the whiskers.

To compare, how selective the spike response magnitude was to direction, a direction selectivity index (DSI) was calculated as the percent decay in amplitude (*A*) from the preferred direction (the direction that evoked the highest average response) divided by the decay constant. In the non-linear fit used to analyze the normalized response to different whisker directions, the value for the preferred direction is 1 (“top” value) and the end value (at 180°) is the “bottom” value. *A* = “top” minus “bottom” value of the fitted curve ^∗^ 100. Tau = time constant of the one-phase fit exponential. Direction selectivity index: DSI = *A*/tau (c.f. Wilent and Contreras, 2005b). Latency to first spike (Figures 3C,D) was calculated separately for each cell as the median first spike latency. The first spike in each sweep was defined as the first spike with a latency from whisker deflection onset of no less than 5 ms, and no more than 100 ms. In our whisker stimulation paradigm, whiskers were deflected four times at 4 Hz every 5 s. The whisker-evoked response was calculated during a 100 ms window following the onset of each whisker deflection. The response to the fourth stimulation, in the 4 Hz train, calculated in a 100 ms time window, intermingled with rebound activity induced after the offset of the 470 nm light, here occurring at the same time as this fourth stimulation (Pauzin and Krieger, 2018). Spikes resulting from the stimulation and the ones from the rebound cannot be fully separated (see Figure 7D), therefore we only report the response to the three first stimulations. The “response probability,” was calculated as a binary measure of how likely the cell was to spike after a whisker deflection. If one or more spikes occurred in a 100 ms window following the onset of the whisker deflection, a value of one was scored, and if no spikes were detected the value was zero. In thalamus the whisker-evoked response was calculated during a 50 ms window following the onset of each whisker deflection. The effect of L6-Ntsr1 activation on the thalamic responses (50 ms time window) was tested individually using a 95% confidence interval for Poisson count (Chi-square distribution), calculated in Excel 2010 using the function ‘CHI2.INV’ or in Excel 2016 using ‘CHISQ.INV’ on the responses in Hertz. The effect of the photo-stimulation was considered significant when the total number of spikes during the optogenetic light stimulation was outside the 95% confidence interval of that for the control condition.

To determine the effect of the optogenetic modulation on spontaneous spiking in cortical excitatory and inhibitory cells, each cell was tested individually using the 95% confidence interval for Poisson count (Chi-square distribution). Spontaneous spiking in excitatory and inhibitory cells was, in control condition, in Ntsr1-cre animals summed over the 300 ms immediately preceding the onset of the optogenetic light (50 sweeps × 300 ms = 15 s in total) and compared to the activity during the 300 ms light pulse. In Gad2-cre animals spontaneous activity, in control condition, was summed over the 320 ms immediately preceding the onset of the optogenetic light (40 sweeps × 320 ms = 12.8 s) and compared to the activity with light stimulation [four light pulses each 80 ms long (= 320 ms), summed over 40 sweeps]. Light stimulation (Ntsr1: single 300 ms pulses: Gad2: four 80 ms pulses at 4 Hz) was repeated every 5 s. An index representing the photo-stimulation effect was calculated as: Opto-Index (OI) = (No-Nc)/(No + Nc). With Nc = number of spikes recorded in control condition; No = number of spikes recorded during photo-stimulation for the same duration. Index varies between −1 and 1, with −1 meaning that with optogenetic activation, of either L6-Ntsr1 cells or Gad2-cells, the recorded cell completely stopped spiking. Cells where the effect was not significant [using a 95% confidence interval for Poisson count (Chi-square distribution)] were scored with an Opto-Index = zero (Supplementary Figure S2). Outliers were identified using the ROUT (Robust regression and Outlier removal) method (with Q = 1%), and the extra-sum-of-squares F test was used to test the DSI curves, both methods used as implemented in GraphPad Prism (GraphPad Software, San Diego, CA, United States). Data are presented as mean ± SEM.

## Results

### Photostimulation of L6-Ntsr1 Neurons

To investigate the contribution of L6-Ntsr1 corticothalamic cells to sensory processing of tactile information, the effect on directional selectivity was investigated in L4 and L5 excitatory cells. Directional selectivity refers to the property that in some cells the whisker-evoked response is dependent on the direction in which the whisker is deflected. *In vivo* electrophysiology recordings were performed in a transgenic mouse line (Ntsr1-cre) with *cre*-labeled L6 cells (Gong et al., 2007; Olsen et al., 2012; Kim et al., 2014; Mease et al., 2014; Velez-Fort et al., 2014; Crandall et al., 2015; Denman and Contreras, 2015; Crandall et al., 2017; Guo et al., 2017; Pauzin and Krieger, 2018). Virus mediated expression of channelrhodopsin-2 (ChR2-mCherry) in the barrel cortex showed bright fluorescence from L6 somata and neuropil (Figure 1A). As has been previously shown in somatosensory cortex (Kim et al., 2014; Mease et al., 2014; Pauzin and Krieger, 2018), neuronal somata expressing mCherry were restricted to L6 ensuring that optical stimulation of cortex specifically modulated the L6-Ntsr1 cell population. These cells make both cortico-cortical (including projections to infragranular interneurons) and cortico–thalamic projections, predominately to ventroposteromedial thalamus (VPM) and to a lesser extent in the posterior medial nucleus (POm), and also project to the thalamic reticular nucleus (Lee et al., 2012; Mease et al., 2014; Chevee et al., 2018). To verify that photoactivation could evoke single-unit spikes, we made juxtacellular recordings from single ChR2-expressing L6 neurons (*n* = 3 cells, three animals; Figure 1B; see also Pauzin and Krieger, 2018) while applying blue light (470 nm) to the cortical surface via an optical fiber. In the example shown in Figure 1B a low light intensity was used (0.2–0.6 mW). Higher light intensities were used to determine if the cell was a L6-Ntsr1 cell or not (see section Materials and Methods). Increasing the light intensity, decreased response latency (Supplementary Figure S1). We have previously shown (Pauzin and Krieger, 2018) that *in vivo* photoactivation of L6-Ntsr1 CT cells in somatosensory barrel cortex reduce cortical activity in other cortical cells via activation of inhibitory interneurons (Figure 1C and Supplementary Figure S1). In the present study a relatively low light intensity was used to ensure that the cortical activity was not reduced to a level where all responses would disappear, irrespective of whisker stimulus strength. With a low light intensity (0.2 – 0.6 mW), spiking in L6-Ntsr1 cells increased from 0.04 ± 0.01 Hz to 7.75 ± 6.6 Hz (*n* = 3, three animals). In most (22 of 25) of the L4 and L5 cells spontaneous activity either decrease or did not change, and it increased in only 3 cells (Supplementary Figure S2). The average Opto-Index was −0.25, meaning that the decrease was 25% of the total number of spikes (sum of control and with optogenetic stimulation).

### Photostimulation of Gad2 Positive Neurons

Evidence suggest that activation of L6-Ntsr1 CT cells activates a sub-set of GABAergic interneurons (Olsen et al., 2012; Bortone et al., 2014; Kim et al., 2014; Guo et al., 2017). The effect on directional selectivity when recruiting a specific GABAergic microcircuit via L6-Ntsr1 CT activation, was compared with a general, unspecific activation of GABAergic interneurons. The Gad2-cre mouse line was used to target ChR2-expression to GABAergic interneurons, to thus achieve a non-specific activation of GABAergic interneurons (Taniguchi et al., 2011; Harris et al., 2014). Virus mediated expression of ChR2-mCherry in the barrel cortex showed bright fluorescence from somata and neuropil in all layers of barrel cortex (Figure 1D and Supplementary Figure S3; Pauzin, 2018). To verify that low intensity photo-activation could evoke single-unit spikes, juxtacellular recordings were done from single ChR2-expressing GABAergic cells (Figure 1E) while applying blue light (470 nm; 0.2 – 0.6 mW) to the cortical surface via an optical fiber (400 μm in diameter). Having established that photoactivation could reliably activate GABAergic interneurons, *in vivo* juxtacellular recordings were done from excitatory cells to compare the effect of L6-Ntsr1 activation and photoactivation of Gad2-cre expressing GABAergic interneurons on the spontaneous activity of excitatory cells (Figure 1F); this was necessary in order to have a comparable level of inhibition when comparing the effect on directional selectivity. Photoactivation of ChR2-expressing GABAergic interneurons (will be referred to as “Gad2-activation”) caused in most (26 of 29) of the L4 and L5 cells either a decrease or no change spontaneous spiking, and it increased in only three cells (Supplementary Figure S2). The average Opto-Index was −0.30, and thus similar (Mann–Whitney test, *p* = 0.2973) to the −0.25 measured for L6-Ntsr1 activation.

### Directional Selectivity in Layer 4 and 5 Excitatory Cells

**FIGURE 2:**
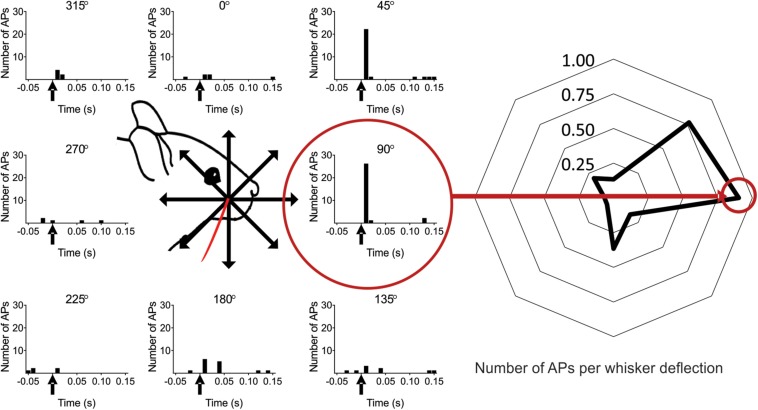
Scheme of the experimental protocol. **(Left)** The whisker was deflected in eight different directions separated by 45°. In this example the whisker was deflected in total 25 times in each of the eight directions (five deflections, five complete turns). The whisker deflection time is at time zero (marked with an arrow in the PSTH). The red circle shows the preferred direction (PD), defined as the direction in which the recorded neuron responded with on average the most spikes per whisker deflection (calculated in a 100 ms time window) (0° is here defined as the straight upward deflection angle. Note: in subsequent figures the *preferred direction* is defined as zero degrees, unless stated otherwise). **(Right)** The data from the left panel plotted as the average number of spikes per whisker deflection (WD) for each angle [100 ms time window, from angle 0° (angle vertical up) to 315°]: 0.13, 0.77, 0.90, 0.17, 0.37, 0.07, 0.07, 0.20 spikes per WD.

**FIGURE 3:**
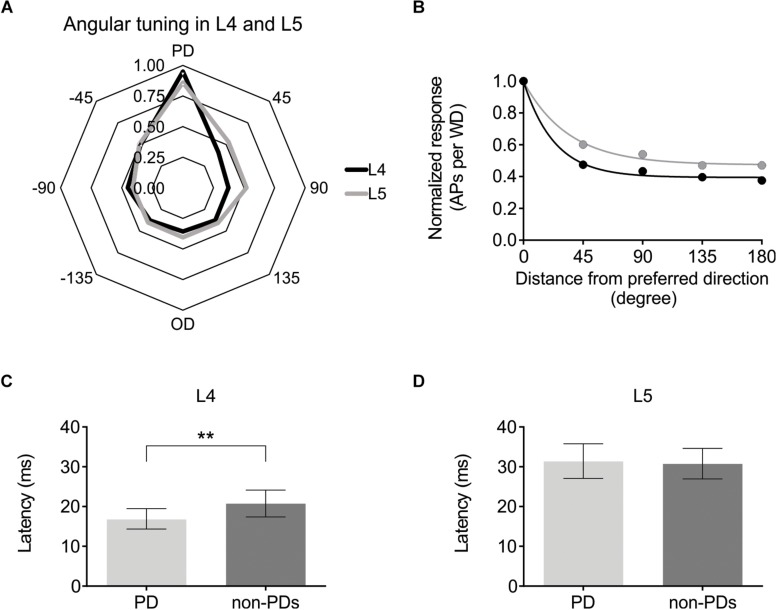
Angular direction is encoded in both L4 and L5 pyramidal cells, albeit with different strength. **(A)** Averaged whisker-evoked response [WER; number of spikes in 100 ms time window after whisker deflection (WD)] for each direction. Preferred Direction (PD) = direction that evoked the most APs for that cell. [For **(A–D)** L4: *n* = 25 cells; L5: *n* = 24 cells]. **(B)** Same data as in **(A)**, now plotted as distance from PD. 45° = average WER of +45 and −45 degrees WD. The larger direction selectivity index (DSI) for L4 indicates that L4 cells are on average more direction selective. (L4: DSI = 2.60; L5: DSI = 1.54). The larger the difference is between the *y*-coordinate at *x* = 0, and the last point (*x* = 180°), corresponding to the plateau value, and the smaller the time constant (meaning a more rapid decay), the larger the DSI. [In **(A,B)**, the cells PD is set to 0°. 45, 90 etc. degrees are thus relative to that cell’s PD. L4 data black line; L5 data gray line]. **(C)** First spike median latency for the PD and for the seven non-PD directions for 22 L4 excitatory cells in control condition in both mouse lines (Ntsr1 and Gad2) (Non-PDs: 20.8 ± 3.4 ms; PD: 16.9 ± 2.6 ms; *p* = 0.0053, paired Wilcoxon test). **(D)** First spike median latency for the PD and for the seven other directions (non-PDs) for 23 L5 excitatory cells in control condition in both mouse lines (Ntsr1 and Gad2) (Non-PDs: 30.8 ± 3.8 ms; PD: 31.4 ± 4.3 ms; *p* = 0.6010, paired Wilcoxon test). ^∗∗^*p* < 0.01.

Having established that optogenetic activation can be used to modulate cortical excitability either via ChR2-expressing L6-Ntsr1 cells or GABAergic interneurons, the effect on sensory processing of directional information was investigated. Firstly, the directional selectivity was determined in the control case, without optogenetic modulation. Whisker-evoked spiking was measured in response to deflections of the whisker in eight different directions (one full turn with 45° difference between the eight directions; Simons, 1983). For each cell, the direction that evoked the highest average number of spikes was called “preferred direction” (PD; Figure 2). In a polar plot of the whisker-evoked responses to different directions, both layer 4 and 5 excitatory cells [L4: *n* = 25 (13 cells from 12 Ntsr1 animals and 12 cells from 6 Gad2 animals); L5: n = 24 (12 cells from each mouse line (same animals as the L4 data))] show a typical “drop shape” (Figure 3A) indicative of angular tuning (Kida et al., 2005; Wilent and Contreras, 2005a; Puccini et al., 2006; Bale and Petersen, 2009; Vilarchao et al., 2018). Calculating the whisker-evoked response individually for each L4 cell (*n* = 25) shows that on average the max response was 142% greater (range: 29 – 300%) to the PD than to the response averaged over all other directions. The same calculation for each L5 cell (*n* = 24) shows that on average the max response was 112% greater (range: 29 – 430%) to the preferred direction than to the response averaged over all other directions. In the recorded excitatory cell sample, the direction that was the PD appeared to be equally distributed over the eight tested directions (Supplementary Figure S4). Figure 3B shows the whisker-evoked responses normalized to the PD response, and plotted against the distance (in degrees) from the PD. To quantify how the whisker-evoked response (WER) decreased compared to the PD response, a direction selectivity index (DSI) was calculated (see section Materials and Methods and Wilent and Contreras, 2005b). The larger DSI for L4 excitatory cells (DSI = 2.60) compared to L5 excitatory cells (DSI = 1.54) indicates that excitatory cells in L4 are more direction selective than those in L5, i.e., the difference between the PD response compared to the other directions is larger for L4 excitatory cells; this is in line with the fact that selectivity probably originates from the anatomically precise convergence of thalamic inputs (Wilent and Contreras, 2005b; Bruno and Sakmann, 2006).

Further evidence that excitatory cells in L4 are more direction selective than L5, can be seen when plotting the first spike latency of the preferred direction (PD) against the first spike latency of all other directions (Figures 3C,D). In L4, the median first spike latency for the PD is shorter compared to all other directions, which is not the case for L5. (Layer; n; latency of PD; latency of all except PD; Wilcoxon test *p*-value) (L4; *n* = 22; 16.9 ± 2.6 ms; 20.8 ± 3.4 ms; *p* = 0.0053) (L5; *n* = 23; 31.4 ± 4.3 ms; 30.8 ± 3.8 ms; *p* = 0.6010). Three cells in L4 and one in L5 were excluded from the latency analysis since they were identified as outliers (using the ROUT method): cell; latency of PD; latency of all except PD: (L4 cell-1: 72.4 ms; 82.1 ms); (L4 cell-2: 49.4 ms; 19.0 ms); (L4 cell-3: 41.5 ms; 21.2 ms); (L5 cell-1: 77.4 ms; 31.3 ms).

### Activation of Different GABAergic Circuits Has Different Effects on Directional Selectivity

**FIGURE 4:**
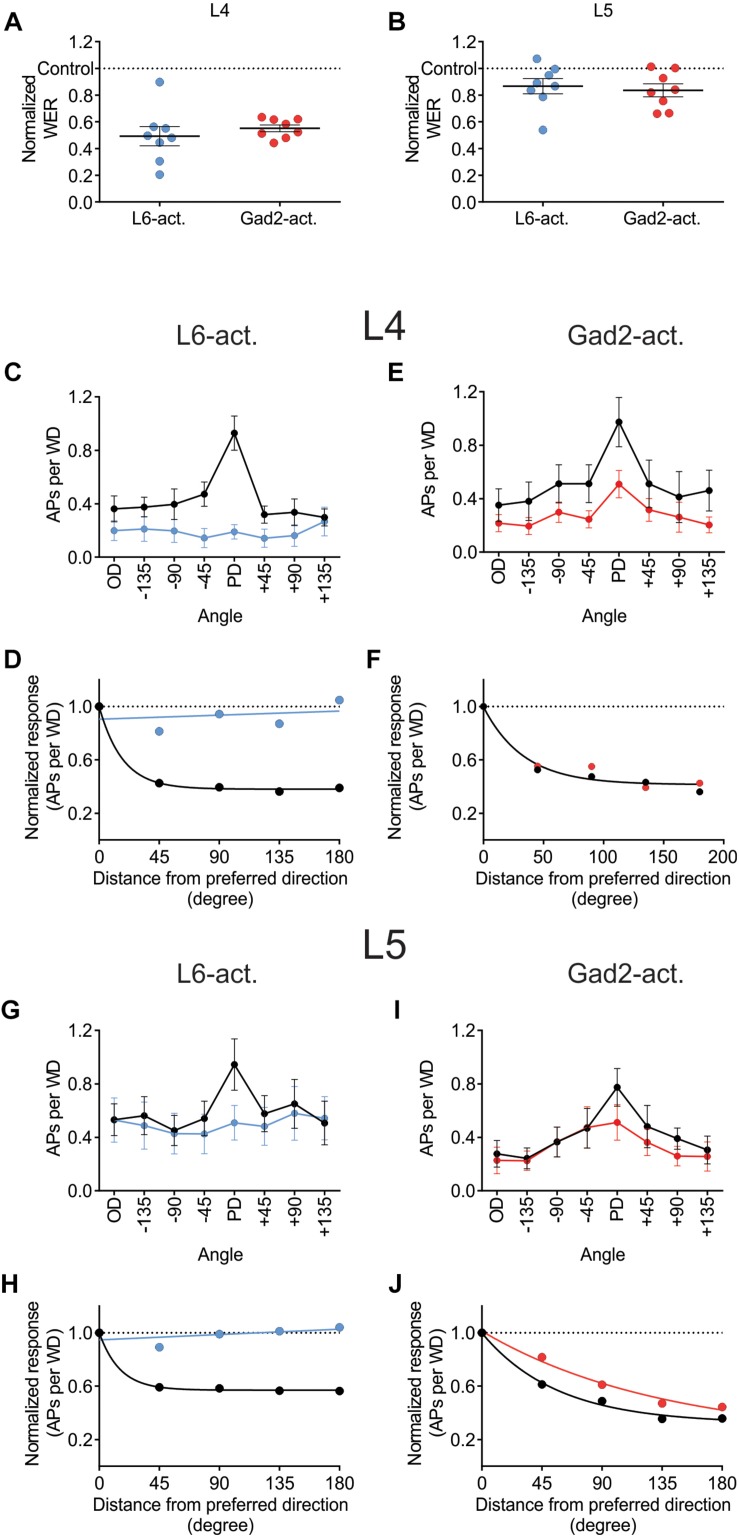
Different effect on angular tuning depending on which GABAergic circuit that is activated. **(A,B)** Normalized decrease of the WER (the WER in control is 1) with photo-activation of L6-Ntsr1 cells or GABAergic interneurons. On average (averaged over all eight directions) the photo-activation decreased the WER with the same strength for both layers in both mouse lines [L4: L6-Ntsr1 act. Vs. Gad2-act., *t*(14) = 0.7674, *p* = 0.4556, unpaired *t*-test; L5: L6-Ntsr1 act vs. Gad2-act., *t*(14) = 0.4179, *p* = 0.6824, unpaired *t*-test]. One data point for each of the eight directions. Number of cells, is for all panels in Figure 4: L4: Ntsr1: *n* = 13, Gad2: *n* = 12; L5: Ntsr1: *n* = 12, Gad2: *n* = 12. **(C)** Averaged WER for L4 excitatory cells in Ntsr1-cre animals. In the L6-Ntsr1 condition, the WER is independent of the whisker deflection direction [interaction: *F*(7,84) = 9.085, *p* < 0.0001; two-way ANOVA with repeated measures on both factors; a test for interaction that is statistically significant, indicates that the effect of the treatment (difference between control and light activation) differs between whisker deflection angles]. **(D)** A different way to show the result from **(C)**, is to plot the same data as normalized whisker-evoked response (WER) with distance (in degrees) from the preferred direction (set at 0°). Direction selectivity index (DSI; see Materials and Methods) for control condition is 3.57 and with L6-Ntsr1 activation zero. **(E)** Averaged WER for layer 4 excitatory cells in Gad2 animals for each condition and each direction. Interaction: *F*(7,77) = 1.215, *p* = 0.3046; two-way ANOVA with repeated measures on both factors. The lack of interaction indicates that light activation affects the WER similarly for all whisker deflection angles. **(F)** Progression of the normalized WER with distance (in degree) from the preferred direction. DSI = 1.75 for both conditions. **(G)** Averaged WER for L5 excitatory cells in Ntsr1 animals for each condition and each direction. In the L6-Ntsr1 activation condition, the WER decreased more for the PD compared to all other directions [interaction: *F*(7,77) = 2.134, *p* = 0.0497; two-way ANOVA with repeated measures on both factors]. **(H)** Normalized WER plotted against distance (in degree) from the preferred direction. DSI control = 2.80, DSI L6-Ntsr1 act. = zero. **(I)** Averaged WER for L5 excitatory cells in Gad2 animals for each conditions and each direction [Interaction: *F*(7,77) = 2.375, *p* = 0.0298; two-way ANOVA with repeated measures on both factors]. **(J)** Progression of the normalized WER with distance (in degree) from the preferred direction. DSI control = 1.20; DSI Gad2-act. = 0.59.

Juxtacellular recordings were made from excitatory cells in layers 4 and 5 of barrel cortex while moving the principal whisker in eight different directions with or without photo-activating either L6-Ntsr1 cells, causing indirect activation of infragranular FS-GABAergic interneurons, or direct activation of GABAergic cells (Gad2-cre mouse line). The whisker-evoked response (spikes/100 ms) was measured in 49 excitatory cells (Ntsr1: 13 L4 and 12 L5 cells; Gad2: 12 L4 and 12 L5 cells). Both L6-Ntsr1 and Gad2 photo-activation caused a significant reduction of the whisker-evoked response. Notably, in both mouse lines, photo-activation caused a stronger decrease in L4 than in L5 for the same light intensity; in line with Pauzin and Krieger (2018) for the L6-Ntsr1 activation condition (Figures 4A,B). Averaged over all eight directions, the whisker-evoked response in L4 decreased 45% from 5.14 ± 0.07 Hz in control condition to 2.81 ± 0.04 Hz with Gad2-activation. Similarly, with L6-Ntsr1 activation the whisker-evoked response decreased on average 57% in L4 from 4.35 ± 0.07 Hz in control condition to 1.89 ± 0.01 Hz with L6-Ntsr1 activation. Both types of stimulation (L6-Ntsr1 act. or Gad2 act.) thus induced a similar strength of inhibition (comparing percent decrease) (unpaired *t*-test, *p* = 0.4556, Figure 4A). The whisker-evoked response in L5 decreased 19%, from 4.13 ± 0.06 Hz in control condition to 3.35 ± 0.04 Hz with Gad2-activation. Similarly, in L5, the whisker-evoked response decreased 16%, from 5.96 ± 0.05 Hz in control condition to 4.98 ± 0.02 Hz in L6-Ntsr1 activation condition. Both GABAergic circuits thus induced a similar strength of inhibition (percent decrease) (unpaired *t*-test, *p* = 0.6824, Figure 4B). This means that the average decreasing effect (averaged over all directions) induced by the optogenetic photoactivation protocol is similar between the two mouse lines; it does, however, not imply that the relative effect was the same for all tested angles, and indeed this was not the case.

Photo-activation of the L6-Ntsr1 cells decreased the whisker-evoked response relatively more when the whisker was deflected in the PD [interaction: *F*(7,84) = 9.085, *p* < 0.0001; two-way ANOVA with repeated measures on both factors; Figure 4C]; this leads to a normalization of the responses, abolishing directional preference in the L4 excitatory cells (DSI for control condition = 3.57, and for L6-Ntsr1 photoactivation = 0; Figure 4D). A DSI equal to zero indicates that the data can be represented by a horizontal line as plotted in Figure 4D and thus the cells display no directional selectivity. For the non-specific GABAergic activation (Gad2-cre mice), the whisker-evoked response in L4 decreased equally (in percentage) for all directions [Figure 4E; interaction: *F*(7,77) = 1.215, *p* = 0.3046, two-way ANOVA with repeated measures on both factors]. This leads to a decreased, but preserved, direction selectivity in the L4 excitatory cells. The direction selectivity index is the same for both conditions (DSI = 1.75; Figure 4F; one curve fits for both data sets using the extra-sum-of-squares *F* test, *p* = 0.1932).

In L5, similar to that found for L4 excitatory cells, L6-Ntsr1 activation lead to a loss of directional selectivity (DSI control = 2.80, and with L6-Ntsr1 act. DSI = 0; Figures 4G,H), whereas with the non-specific GABAergic activation (Gad2-cre mice), the DSI is reduced, but still not zero (DSI control = 1.20, and with Gad2-activation DSI = 0.59; Figures 4I,J). A different way to analyze the same data is to calculate a summed vector and analyze how the vector direction changes with light activation (Supplementary Figure S5); this analysis showed that the vector direction changed significantly more, i.e., direction selectivity decreased more, with L6-Ntsr1 activation.

### A Reduced Response Probability Leads to the Decreased Directional Selectivity With L6-Ntsr1 Photoactivation

**FIGURE 5:**
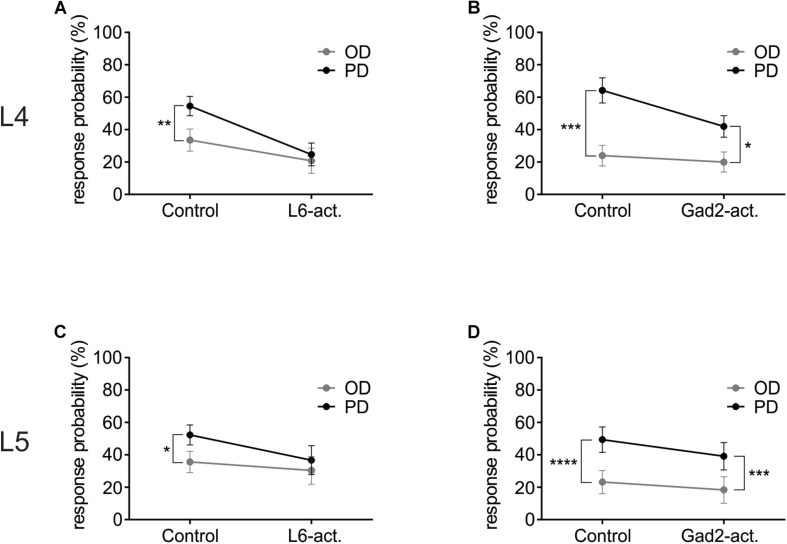
L6-Ntsr1 photoactivation, but not Gad2-activation caused the cells to be equally likely to respond to the preferred (PD) as the opposite direction (OD, 180° from the PD). **(A)** In L4 excitatory cells, with L6-Ntsr1 activation (*n* = 13), there was no difference in the response probability between PD and OD whisker deflection. **(B)** In contrast, for Gad2-activation (*n* = 12) the difference between PD and OD persisted even with photoactivation. **(C,D)** A similar result was found for L5 excitatory cells, where L6-Ntsr1 activation (*n* = 12), but not Gad2-activation (*n* = 12) abolished the difference in response probability between the PD and OD response. Stars (^∗^) refer to result from repeated measure two-way ANOVA and Sidak’s multiple comparison test.

To investigate how L6-Ntsr1 activation alters the spiking statistics such that direction selectivity is lost in L4 and L5 excitatory cells, a complementary analysis was done on the whisker-evoked responses. The change in response probability (number of whisker deflections that evoked at least one spike/100 whisker deflections, Figure 5) was different depending on which GABAergic circuit was activated. The largest difference in terms of average spikes triggered per whisker deflection is between the preferred direction and its opposite direction (180° to the PD); the analysis was therefore done on these two whisker deflection angles. In L4, in control condition in both mouse lines the response probability was greater if the whisker was deflected in the cells preferred direction than if it was deflected in the opposite direction (Figures 5A,B and Supplementary Figure S6). The difference in response probability to a PD or OD (opposite direction) stimulation is abolished with L6-Ntsr1 activation (Figure 5A). This means that with L6-Ntsr1 activation the detectability (number of events that evoke spiking) is no longer influenced by the direction in which the whisker was deflected [response probability (RP) in control: PD: 55 ± 6.0%; OD: 34 ± 6.9%; Sidak’s multiple comparison test used for all response probability calculations: p = 0.0022]; (RP with L6-Ntsr1 activation: PD: 25 ± 7.0%; OD: 21 ± 7.7%; *p* = 0.3092). In contrast, the difference in response probability between PD and OD stimulation was not blocked with a non-specific GABAergic activation (Figure 5B). This means that the probability that a whisker deflection evokes spiking in a cell, is still dependent on the angle in which the whisker was deflected (RP in control: PD 64 ± 7.7%, OD 24 ± 6.4%; *p* = 0.0001; RP with Gad2-activation: PD 42 ± 6.6%, OD 20 ± 6.2%, *p* = 0.0126). The same result was found for the excitatory L5 cells [Figures 5C,D and Supplementary Figure S6; (RP in Gad2-cre mice in control: PD 49 ± 7.8%, OD 23 ± 7.2%, *p* < 0.0001; RP with Gad2-activation: PD 39 ± 8.4%, OD 18 ± 8.2%, *p* = 0.0002); (RP in Ntsr1-cre mice in control: PD 52 ± 6.2%, OD 36 ± 6.5%, *p* = 0.0459; RP with L6-Ntsr1 activation: PD 37 ± 8.9%, OD 30 ± 8.6%, *p* = 0.6430)].

### L6-Ntsr1 Activation Did Not Affect Direction Selectivity in VPM

**FIGURE 6:**
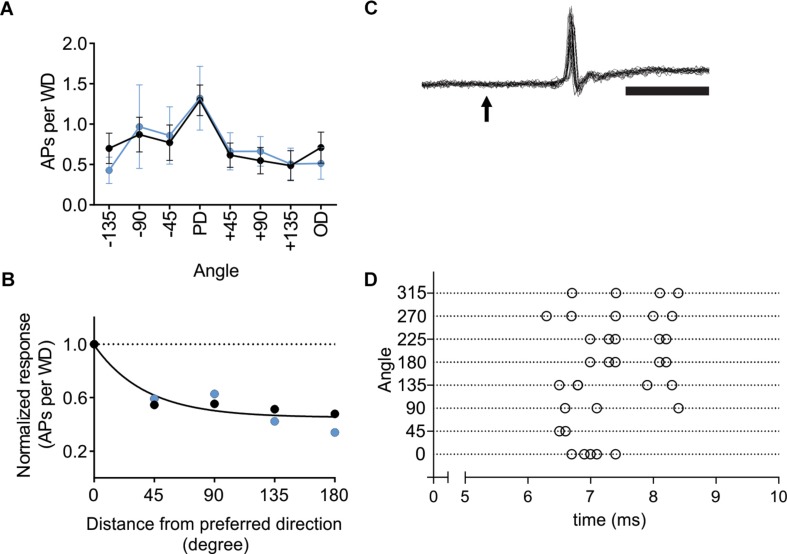
In VPM thalamus L6-Ntsr1 activation did not change direction selectivity. **(A)** Averaged whisker-evoked response (WER) for VPM cells (*n* = 8, two animals) in Ntsr1-cre animals in control (black line) and with L6-Ntsr1 photoactivation (blue line) measured for eight different directions. No significant difference between control and L6-Ntsr1 activation condition for any of the tested angles [Interaction: *F*(7,102) = 0.1586, *p* = 0.9924, two-way ANOVA]. **(B)** The WER normalized to the response for the PD. The WER showed the same dependence on whisker deflection angle in control and with L6-Ntsr1 activation (one curve fits for both data, extra-sum-of-squares *F* test, *p* = 0.4924). Direction selectivity index (DSI) was 1.42 for both conditions. **(C)** An example of a juxtacellular VPM recording. The arrow indicates the time point of whisker deflection. In this example, the whisker was deflected five times in four different directions, thus 20 sweeps are overlaid. Notably, there is no jitter in the latency of the first spike and response probability was 100%. Scale bar 5 ms. **(D)** Raster plot showing an example of the first spike latency for the eight different whisker deflection angles. Each angle was tested five times. The VPM cell in **(D)** is a different from that in **(C)**.

Activation of L6-Ntsr1 CT cells changes whisker-evoked activity not only in cortex but also adaptation in thalamus (Mease et al., 2014; Supplementary Figure S7). To investigate if L6-Ntsr1 activation abolished direction selectivity already in the thalamic VPM nucleus, in a new set of experiments, the directional selectivity was determined for VPM cells with and without L6-Ntsr1 activation. The light intensity of the optogenetic LED, and the whisker deflection protocol were the same as for the cortical recordings. The whisker-evoked response (quantified as spikes/50 ms; whiskers deflected every 5 s) was measured in eight VPM cells. The effect of L6-Ntsr1 photo-activation on the whisker-evoked response in the PD varied between cells (increase: *n* = 1; decrease: *n* = 2; no significant change: *n* = 5; chi-square test for a Poisson distribution). Averaging over all eight cells, L6-activation did not change the PD response or the direction selectivity curve [Figure 6A; Interaction: *F*(7,102) = 0.1586, *p* = 0.9924, two-way ANOVA]. The DSI is the same for both conditions (DSI = 1.42, Figure 6B; one curve fits for both data using the extra-sum-of-squares *F* test, *p* = 0.4924). Furthermore, the latency of the first spike was, at least in this cell sample (cf. Storchi et al., 2012), not affected by the direction in which the principal whisker had been deflected (comparing PD to all other directions, *n* = 8 cells from two Ntsr1-animals, non-PDs: 10.68 ± 2.2 ms; PD: 13.44 ± 3.1 ms; Wilcoxon test, *p* = 0.1250; see Figures 6C,D for an example showing that 1st spike latency did not fluctuate).

### Adaptation and Cortical Inhibition

**FIGURE 7:**
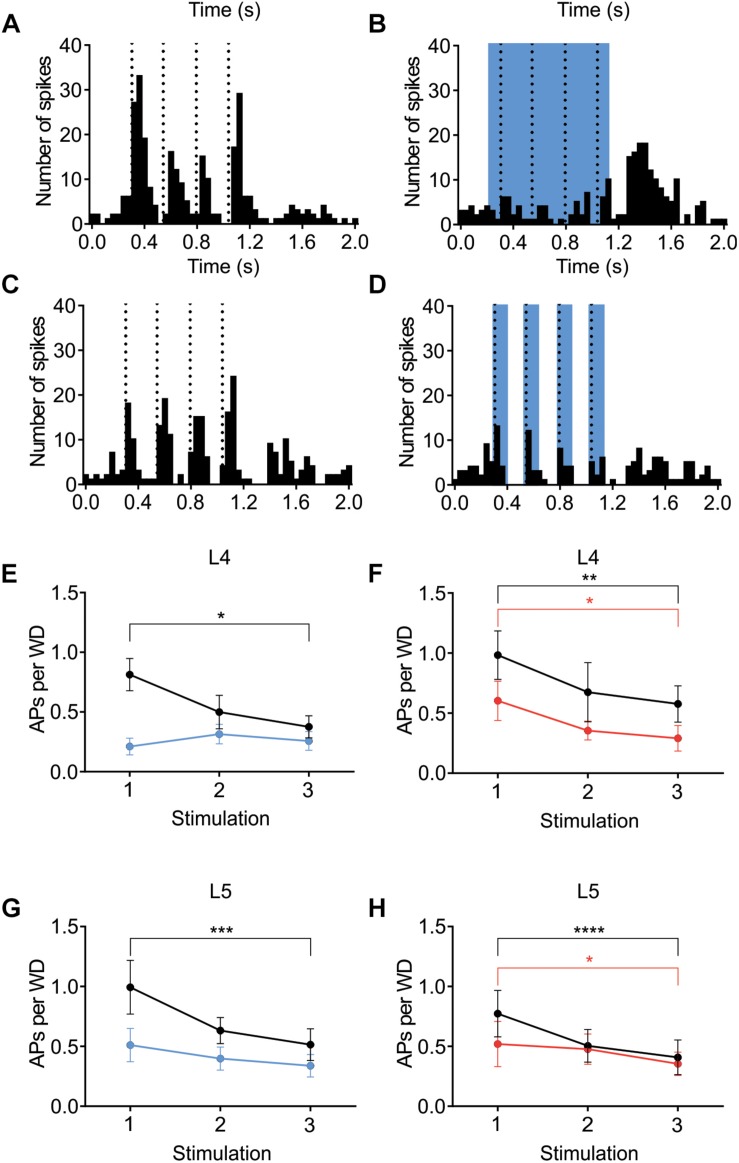
Adaptation to repetitive, 4Hz whisker stimulation in the preferred direction. Examples **(A–D)** of the effect of light stimulation (blue) on a train of 4 whisker deflections (each 25 ms ramp-and-hold) at 4Hz repeated every 5 s. **(A,B)** whisker-evoked responses (dotted lines) recorded in a L4 cell in a Ntsr1-animal and **(C,D)** a L4 from a Gad2-animal (protocol as outlined in Materials and Methods and main text). **(E)** In L4 the response adapted in control, but not when L6-Ntsr1 cell were activated (*n* = 10) (control: *p* = 0.0302, L6-Ntsr1 activation: *p* = 0.9867; Table 1). *y*-axis shows the average number of spikes evoked by each whisker deflection. **(F)** Layer 4 cells adapted in both control and with Gad2 photoactivation (*n* = 11) (control: *p* = 0.0026; Gad2-activation: *p* = 0.0194). **(G,H)** A similar result was found for L5 excitatory cells with adaptation eliminated with L6-Ntsr1 activation (*n* = 10), but still present with Gad2 activation (*n* = 8). (control *p* = 0.0002, L6-Ntsr1 activation *p* = 0.2119; control *p* < 0.0001, Gad2-activation condition: *p* = 0.0343). *p*-values are adjusted for multiple comparisons from the Sidak’s multiple comparisons test (comparing 1st stim. to 3rd stim.) following two-way ANOVA repeated measure on both main factors. ^∗^*p* < 0.05, ^∗∗^*p* < 0.01, ^∗∗∗^*p* < 0.001, ^∗∗∗∗^*p* < 0.0001.

The contribution of L6 CT cells to sensory processing can vary depending on the sensory input characteristics (Temereanca and Simons, 2004; Li and Ebner, 2007; Mease et al., 2014; Crandall et al., 2015; Denman and Contreras, 2015; Guo et al., 2017; Pauzin and Krieger, 2018). An object detection task, for example, requires fewer touches, whereas a more complex task where object characteristics are analyzed would require more touches. To analyze if the effect of cortical inhibition via activation of L6 CT differs from that of Gad2-cell activation, not only during a “detection” task mimicked with the low frequency stimulation reported above, the whiskers were stimulated with a train of 4 Hz whisker deflections, to mimic a more complex tactile processing task. Figures 7A–D shows examples from L4 excitatory cells in an Ntsr1-animal and Gad2-animal. Figure 7 shows the averaged whisker-evoked response for the first three stimulations in the 4Hz whisker deflection train. Furthermore, plotted are only cells [*n* = 39 of 49 cells, sampled from in total 18 animals (6 Gad2 animals and 12 Ntsr1 animals)] displaying adaptation in control (ratio [3rd stim. response/1st stim response] < 1). Cells showing facilitation in control were not included in this calculation (ratio [3rd stim. response/1st stim response] > 1) [*n* = 10 out of 49; (L6-Ntsr1 activation: three L4 cells; two L5 cells; Gad2-activation: one L4 cell; four L5 cells)]. In L4 and L5, responses in control adapted and this adaptation was preserved in both layers with the general GABAergic activation, but was lost, as previously reported (Pauzin and Krieger, 2018), with the L6-Ntsr1 activation (Figure 7 and Table 1). L6-Ntsr1 activation, before the first whisker deflection, thus switching cortex to an already “adapted mode” where whisker-evoked responses are not adapted further.

**TABLE 1:** Related to Figure 7.

**Mouse line**	**Layer**	**Condition**	**1st stim APs/WD**	**3rd stim APs/WD**	**Statistics**
Ntsr1 animals	L4 (*n* = 10)	Control	0.81 ± 0.14	0.38 ± 0.09	0.0302
		Opto.	0.21 ± 0.07	0.26 ± 0.08	0.9867
	L5 (*n* = 10)	Control	0.99 ± 0.22	0.51 ± 0.14	0.0002
		Opto.	0.51 ± 0.14	0.34 ± 0.09	0.2119
Gad2 animals	L4 (*n* = 11)	Control	0.98 ± 0.20	0.58 ± 0.15	0.0026
		Opto.	0.60 ± 0.16	0.29 ± 0.11	0.0194
	L5 (*n* = 8)	Control	0.77 ± 0.19	0.41 ± 0.14	<0.0001
		Opto.	0.52 ± 0.19	0.35 ± 0.10	0.0343

*Effect of Gad2 or L6-Ntsr1 cell activation on adaptation of whisker–evoked responses. “Opto.”: optogenetic activation of either channelrhodopsin expressing L6-Ntsr1 or Gad2 cells. Two-way ANOVA repeated measure on both main factors with *p*-values from Sidak’s multiple comparisons test (comparing 1st stim. to 3rd stim.). Notably, the adaption was lost with L6-Ntsr1 photoactivation (see cells marked gray). APs per WD = Action Potentials per Whisker Deflection. Data presented as mean ± SEM.*

### Direction Selectivity and Interneurons

**FIGURE 8:**
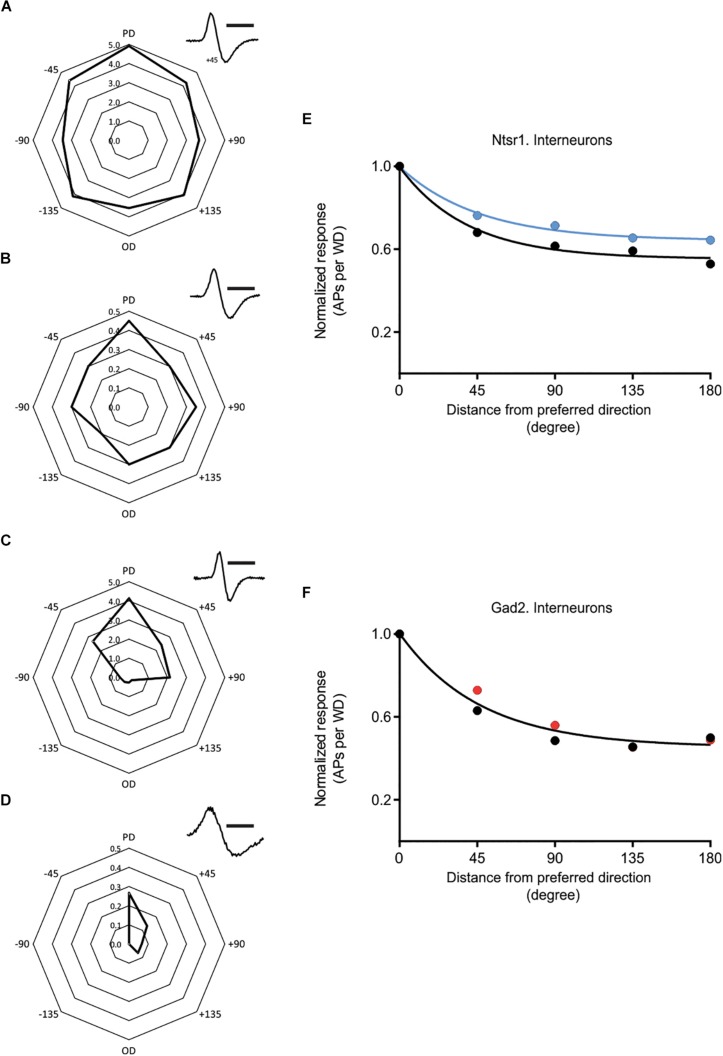
Effect of cortical inhibition on directional selectivity in GABAergic interneurons. Examples of the whisker evoked responses (WER; spikes per whisker deflection, 100 ms time window) in four putative GABAergic cells [identified by spike shape (*inset*)]. Examples of (i) strongly responding cell showing a weak **(A)** or stronger **(C)** orientation tuning, and (ii) less responding interneurons with **(B)** displaying a weak orientation tuning, and **(D)** a stronger angular tuning. **(E)** Normalized progression of the WER with distance (in degree) from the preferred direction. Direction selectivity index (DSI) for control condition is 1.13 and for L6-Ntsr1 activation condition 0.78 (*n* = 5 cells). **(F)** The WER showed the same dependence on whisker deflection angle in control and with Gad2-activation (one curve fits for both data, extra-sum-of-squares *F* test, *p* = 0.2883). DSI was 1.17 for both conditions (*n* = 11 cells). [**(A)** recorded at a depth of 268 μm in a Gad2-cre animal; **(B)** 872 μm, Ntsr1-cre; **(C)** 665 μm, Gad2-cre; **(D)** 168 μm, Gad2-cre]. Polar plots in **(A–D)** shows spikes/whisker deflection. Spike shape, scale bar = 1 ms.

In addition to the above results obtained from excitatory cells, spikes were also recorded from putative GABAergic interneurons. There was a high variability, in control condition, in the directional selectivity (Figure 8). To investigate if the interneuron population changed their directional selectivity, recordings were made from interneurons in Gad2-cre and Ntsr1-cre animals injected with channelrhodopsin. Analyzed were interneurons where light stimulation did not activate the recorded interneuron, but either had no effect or decreased spontaneous spiking. Interneurons activated by light were not used, because in these cells spiking simply increased to all directions with light. The effect of photo-activation was quantified measuring the relative change in spiking (using the Opto-Index calculation). There was no difference, in the effect of photo-activation on spiking, between the interneurons recorded in the Gad2-cre (*n* = 11, 4 animals) and Ntsr1-cre (*n* = 5, 5 animals) animals (comparing the Opto-Index; Mann–Whitney *U* = 27, *p* = 0.9931, data not shown). Interneurons (*n* = 5) recorded in Ntsr1-cre animals, tended to be less directional selective with light stimulation (Figure 8E; control condition: DSI = 1.13, L6-Ntsr1 activation: DSI = 0.78; a single curve cannot fit the normalized decrease). In contrast, in Gad2-animals the directional selectivity appeared not to change with light stimulation (Figure 8F; one curve fits both data sets control and Gad2-activation; extra-sum-of-square *F* test, *p* = 0.2883, DSI = 1.17, *n* = 11 cells).

## Discussion

In this study, we investigated how increased inhibition modulates the angular tuning properties of L4 and L5 excitatory cells. The decrease in cortical activity was achieved either “indirectly” via L6-Ntsr1 photoactivation, which recruits GABAergic interneurons, or directly via photoactivation of GABAergic interneurons. The direct GABAergic activation (channelrhodopsin activation in Gad2-cre mouse line) reduced the whisker evoked response (WER) to a similar degree for all whisker deflection angles, thus the cell’s angular tuning is preserved. In contrast, L6-Ntsr1 activation, leading to the activation of mostly infragranular fast spiking interneurons, reduced the WER in L4 and L5 excitatory cells with different strength depending on the whisker deflection angle. The preferred direction was reduced more, thereby abolishing the angular tuning. Importantly, using a relatively low light intensity to optogenetically activate cells, the effect on spontaneous spiking and the reduction of the averaged WER (all directions) was comparable between the experiments in the Ntsr1-cre and Gad2-cre animals, thus the difference in the effect is most likely due to activation of different circuits rather than simply due to a difference in the global level of inhibition. The decreased directional selectivity with L6-Ntsr1 photoactivation was not caused by changes in the directional selectivity in VPM (Figure 6). Inhibition caused by L6-Ntsr1, but not Gad2 photoactivation, shifted cortical activity to an adapted mode in which sensory adaption was reduced (Figure 7).

Layer 4 excitatory cells were more directional selective than L5 excitatory cells (Figure 3). Furthermore, averaging the whisker-evoked response over all directions, photo-activation leads to a stronger reduction of the whisker-evoked response in L4 compared to L5 in both mouse lines (Figures 4A,B). In L4 the whisker-evoked response to all directions tend to decrease (Figures 4C,E), whereas in L5 it is in particular the preferred direction that is decreased (Figures 4G,I). The stronger decrease in the preferred direction in L5 cells can explain why, when averaging over L5 cells where the whisker is not always deflected in the preferred direction, the decrease in the whisker-evoked response, when photo-activating L6-Ntsr1 cells, was reported to be weaker in L5 compared to the other layers (see Pauzin and Krieger, 2018). It can be noted that when cells in rat barrel cortex were instead binary classified as direction selective or not (according to a stipulated criteria) the proportion of L4 and L5 being direction selective was equal (Khateb et al., 2017).

### Cortical Activity and Directional Selectivity

Previous investigations on how a change in cortical inhibition affects directional selectivity have found that blocking inhibition, with the GABA antagonist bicuculline, results in L4 cells losing their angular tuning, because the response to a whisker deflection in the non-preferred direction increased (Kyriazi et al., 1996). A similar broadening of angular tuning was found in the principal trigeminal nucleus after pharmacological blockade of inhibition (Bellavance et al., 2010). Furthermore, micro-iontophoretic application of GABA sharpened angular tuning (Kyriazi et al., 1996). In the present study, where inhibition was increased by stimulating the GABA releasing cells, a different result was found. Optogenetic activation of the Gad2-expressing cells reduced the response, but angular tuning was maintained. In the present experiments, the optogenetically induced inhibition via activation of Gad2-expressing cortical cells was kept relatively low, to have an inhibition comparable to that induced by L6-Ntsr1 activation. The global application of GABA via micro-iontophoresis undoubtedly causes a different type of inhibition. In the same way, a more global electrical stimulation of layer 6 has been shown to effect the angular tuning in VPM thalamus cells (Li and Ebner, 2007), which is in contrast to that found in the present study with a more selective, and relatively restricted activation of L6-Ntsr1 cells. Importantly, the sharpening of angular tuning curves in VPM cells (Li and Ebner, 2007), was dependent on the stimulation and recording in an aligned barrel column and barreloid, respectively, from cells tuned to the same direction. In the present experiments the viral injection covers more than one barrel column, and the optogenetic activation and subsequent recording are not necessarily aligned, nor can activation be restricted to L6-Ntsr1 cells with the same angular tuning as the recorded VPM unit. The present result show that local changes in cortical excitability can affect angular tuning, and that the change is not inherited from effects on VPM thalamus. It is thus not in contradiction to a differential effect of aligned or non-aligned layer 6 corticothalamic cells on VPM angular tuning. As previously shown (Mease et al., 2014; Pauzin and Krieger, 2018) activation of L6-Ntsr1 cells can increase spontaneous activity and decrease whisker-evoked adaptation in VPM, both mechanisms that contribute (Castro-Alamancos and Oldford, 2002; Chung et al., 2002) to cortex being in an “adapted” mode and thus showing less adaptation. Although the changes in directional tuning was not inherited from thalamus, clearly other aspects of cortical activity can be.

Activation of GABAergic interneurons via L6-Ntsr1 activation, but not via a general activation of Gad2-expressing interneurons, tended to decrease directional selectivity in some interneurons, i.e., those not activated by the photostimulation (Figure 8). Interestingly, it was thus only L6-Ntsr1 driven inhibition that decreased angular tuning in this population, and this could indicate that interneurons driven by L6-Ntsr1 CT activation form separate microcircuits that converge on other cells to exert a more powerful effect. If there is some intrinsic difference in the interneuron population recruited by L6-Ntsr1 activation that can explain the difference to a global non-specific recruitment of interneurons remains, however, to be fully investigated. It should be noted that the comparison (Figure 8) is between interneurons in Gad2 and Ntsr1 mice that were not affected or decreased their activity to optogenetic activation, thus this comparison is between a subset of inhibitory neurons. Furthermore, one interneuron driven by L6-Ntsr1 cells did not appear to show directional selectivity in control condition (Supplementary Figure S8); if this were to be corroborated it indicates that it is rather the temporal precision when these interneurons are activated, rather than angular tuning *per se*, that determines the effect on the angular tuning of excitatory cells. Indeed, *in vivo* electrophysiology recordings from rat somatosensory barrel cortex have shown that for deflections in a cells preferred direction, excitation precedes inhibition (Wilent and Contreras, 2005a).

Since the decrease was specific to activation of GABAergic inhibition via L6-Ntsr1 activation, the decrease in directional selectivity is not simply due to a non-specific increase in inhibition. We interpret the present results such that the contribution of inhibition to directional selectivity in excitatory cells should not be thought of as mainly providing a given background inhibition that is overcome by large short-latency excitation when the whisker deflection is in the preferred direction, but rather that a designated inhibitory microcircuit is involved to mediate the correct temporal relationship between excitation and inhibition. The greatest enemy of excitation is the absence of properly timed inhibition.

### Orientation Tuning in the Visual System Versus Angular Tuning in the Whisker System

In the mouse primary visual cortex, neurons are tuned to respond to gratings of different orientations (Hubel and Wiesel, 1962; Niell and Stryker, 2008; Olsen et al., 2012). Optogenetic activation of L6-Ntsr1 CT cells in the mouse visual cortex did not affect the orientation selectivity index of cortical neurons throughout layers 2/3, 4 and 5 (Olsen et al., 2012). Although some features of L6-Ntsr1 activation appears similar in the whisker and visual system, e.g., reduction of sensory evoked responses in cortex (Olsen et al., 2012; Pauzin and Krieger, 2018), the fine details appear different. Even though neurons in both visual and somatosensory cortex can respond more to sensory stimuli “moving” in a certain direction, visual orientation tuning and tactile angular tuning are two different phenomena.

### Object Detection and Texture Discrimination

Modeling directional selectivity in the whisker system by tuning the synaptic amplitude and latency, it was suggested that directional selectivity is modulated by the frequency of an ongoing stimulus (Puccini et al., 2006). It was argued that directional selectivity may be a feature more prominent for the detection of the object location, and of less importance in tasks requiring multiple whisker touches, such as for texture discrimination (Puccini et al., 2006). During a detection task, here mimicked by the first whisker deflection of the 4 Hz train, the localization of an object is most important whereas the nature of this object (size, shape, texture, etc.) requires additional tactile touches. During a discriminability task (here mimicked by a 4 Hz whisker deflection train), the details about an object (texture, exact form, etc.) can be analyzed rather than the object position; the object has already been detected and now needs to be characterized. With repetitive stimulation the evoked responses get smaller, the response adapts. Sensory adaptation has consequences for the processing of information, and interestingly decreasing cortical activity via different mechanisms have different effects. In both L4 and L5 excitatory cells, the L6-Ntsr1 driven decrease in cortical activity reduced the 1st response to a level were no further adaptation occurred. In contrast, the direct GABAergic activation in L4 reduced all three responses such that the response curve was shifted down, with maintained adaptation. This result could thus indicate that activation of GABAergic interneurons by L6-Ntsr1 cells switches cortex to an adapted mode. A switch to an adapted mode occurring already before the first input reaches cortex and thus additional whisker deflections failed to reduce the response. In L5, the GABAergic effect was similar. A key function of L6-Ntsr1 activation in barrel cortex thus appears to be the modulation of sensory adaptation (Mease et al., 2014; Pauzin and Krieger, 2018). In the whisker system, adaptation to tactile stimuli appears to either worsen or improve sensory processing depending on the behavioral situation (Maravall et al., 2007; Musall et al., 2014; Ollerenshaw et al., 2014; Mohar et al., 2015; Whitmire et al., 2016; Lampl and Katz, 2017). Our prediction is then that stronger L6-Ntsr1 activity – leading to less adaptation – would be beneficial when the frequency of tactile input is high, such as during texture or vibration discrimination. The higher the input frequency/stimulation strength the more difficult it is for an individual cell to follow each input. Thus response probability is decreased for each individual cell, to improve specificity, but the decrease in spiking from an individual neuron is compensated by population coding (Abbott and Dayan, 1999; Petersen et al., 2009; Farkhooi et al., 2013; Liu et al., 2017).

## Data Availability Statement

The datasets generated for this study are available on request to the corresponding author.

## Ethics Statement

The animal study was reviewed and approved by the Landesamt für Natur, Umwelt und Verbraucherschutz, Nordrhein-Westfalen.

## Author Contributions

FP and PK designed the research, analyzed the data, and wrote the manuscript. FP and NS performed the experiments.

## Conflict of Interest

The authors declare that the research was conducted in the absence of any commercial or financial relationships that could be construed as a potential conflict of interest.
