# Supplementary material for: Activation of Corticothalamic Layer 6 Cells Decreases Angular Tuning in Mouse Barrel Cortex
Source: Front Neural Circuits. 2019 Nov 1;13:67. doi: 10.3389/fncir.2019.00067 (PMC6838007; doi:10.3389/fncir.2019.00067)
Supplement: Supplementary file 1 [file Data_Sheet_1.pdf]

Supplementary Material for

Activation of corticothalamic layer 6 cells decreases angular tuning in mouse barrel cortex

François Philippe Pauzin, Nadja Schwarz, Patrik Krieger

Supplementary Figure 1. Related to Figure 1.

Infragranular interneurons activated by direct projections from L6-Ntsr1 cells

In vivo electrophysiology recording from a putative fast-spiking (FS) interneuron (872 µm; same cell as in Fig. 1C) activated by light stimulation of L6-Ntsr1 cells. (*A*) Increasing the light intensity reduced the latency to the first light evoked spiking response. (*B*) Even with the highest light intensity (6.35mW) the latency to the first spiked varied (jitter: 3.5 ms), suggesting that the cell was not directly activated by light, but rather indirectly via light activation of L6-Ntsr1 cells that project to the interneuron. (C) Fast-spiking units (presumably interneurons) were distinguished from regular-spiking units (presumably excitatory cells) based on the spike peak-to-trough duration (y-axis) and on the symmetrical properties of the up and down deflection (integral of the spike shape; x-axis). Based on these variables, cells were classified using the K-means clustering (n = 2 clusters) method. Plotted are the z-scores of the peak-to-trough duration and "integral" from 152 cells (119 classified as excitatory and 33 as inhibitory).

Supplementary Figure 2.

L6-Ntsr1 activation and Gad2-activation decreased the spontaneous activity of excitatory cells with the same strength

To determine the effect of the optogenetic modulation on spontaneous spiking in cortical excitatory cells, each cell was tested individually using a 95% confidence interval for Poisson count (Chi-square distribution, see Materials and Methods). An index representing the photo-stimulation effect was calculated as: Opto-Index (OI) = (No-Nc) / (No+Nc). With Nc = number of spikes recorded in control condition; No = number of spikes recorded during photo-modulation for the same duration. Index varies between -1 and 1, with -1 meaning that with optogenetic activation, of either L6-Ntsr1 cells or Gad2-cells, the recorded cell completely stopped spiking. Cells where the effect was not significant were scored with an Opto-Index = zero. For the excitatory cells found in the Ntsr1 mouse line, most (22 of 25) of the L4 & L5 cells spontaneous activity either decreased or did not change, and it increased in only 3 cells. The average OI was -0.25, meaning that the decrease was 25% of the total number of spikes measured in both control and with optogenetic stimulation. For the excitatory cells recorded in the Gad2-cre line, most (26 of 29) of the L4 & L5 cells spontaneous activity either decreased or did not change, and it increased in only 3 cells. The average OI was -0.30, and thus similar (Mann Whitney test, p = 0.2973) to the -0.25 measured for L6-Ntsr1 activation.

Supplementary Figure 3. Related to Figure 1D.

Gad2-cre expression in mouse barrel cortex

Coronal brain slice showing the expression of AAV-dfloxed-ChR2-mCherry in a Gad2-IRES-cre mouse. The virus was injected at a depth of 0.9 mm below pia. Fluorescently labelled cells were found throughout all layers of barrel cortex.

Supplementary Figure 4. Related to Figure 3.

Distribution of the preferred direction

The number of cells having a given angle as it preferred direction (PD). In this cell sample there was no difference between L4 and L5 with regards to the distribution of PD (L4: n = 25 (Ntsr1 = 13, Gad2 = 12); L5: n = 24 (Ntsr1 = 12, Gad2 = 12), statistics was done on all cells combined for a given angle; Chi-square test, p = 0.5991). Zero degrees is vertical up. Furthermore, there was no difference between L4 and L5 cells with regards to which angle was the preferred direction (PD) (Chi-square test, p = 0.5991). In addition, within each cell group (L4 or L5, respectively) no angle was more often the PD (For both L4 and L5 cells the two-tailed p > 0.7; "Chi-square test for goodness of fit", i.e. the observed distribution, for L4 and L5 respectively, was compared with an expected distribution, which in this case was equal number of cells for each of the eight angles). The low number of cells in each of the eight tested directions makes this type of test only indicative.

Supplementary Figure 5. Related to Figure 4.

Cortical inhibition driven by L6-Ntsr1-activation, but not via non-specific GABAergic activation abolished angular tuning

The result presented in Figure 4 can also be quantified by calculating how much the summed vector direction changes. It is evident from Figure 4 that with cortical inhibition it is not the case that, e.g. all cells shift to a new preferred direction, but rather that directional selectivity is, on average, lost or decreased. Under these conditions the larger the effect of photoactivation on directional selectivity the larger the change in summed vector direction. Firstly, the vector length was calculated as follows: the responses (spikes / whisker deflection (WD), were assumed to be symmetrical, i.e. the whisker-evoked response decreased as a function of the relative angular difference to the preferred directions (an assumption justified by the symmetrical shape of the data plotted in Figure 4; see also (Wilent and Contreras, 2005a)) around the preferred direction (PD), thus in the calculation zero is the preferred direction. 45 deg is the average response for the angle located at +45 and -45 degree from the PD. Same for the angles 90 and 135, and 180 is the OD response. A vector was calculated for each angle (0, 45, 90, 135, 180). The vectors were added, resulting in a summed vector $\bar{a} = \{a_x;\, a_y\}$. The direction θ of this summed vector $\bar{a}$ is $\theta = tan^{-1}\left(\frac{a_y}{a_x}\right)$. The direction of the summed vector changes more after photo-activation of L6-Ntsr1 cells than after Gad2-activation (for a similar strength of inhibition). The mean change in degrees for the summed vector direction after L6-Ntsr1 condition is 38 ± 25 deg (range: 3 to 84) while it is of 17 ± 12 deg (range: 3 to 39) after Gad2-activation (Gad2: n = 12, Ntsr1: n = 13, unpaired t-test, p = 0.0150). The fact that the range of the changes in the summed vector direction after L6-Ntsr1 activation is larger, is in this context representative of a loss in direction selectivity. Data presented as mean ± SD.

Supplementary Figure 6. Related to Figure 5.

Effect of L6-Ntsr1 activation on the detectability of whisker deflection direction

Same data as in Figure 5, but now plotted for each individual cell. For each condition (Control, L6-act., Gad2-act.) and for both L4 and L5, the response probability (see Materials and Methods) to whisker deflection in either the individual neuron's preferred direction or opposite direction (180 deg to the cells preferred direction) is plotted. (*A*, *B*) In L4, in the control condition for both mouse lines, the response probability (RP) is higher when the whisker is deflected in the neuron's preferred direction compared to the opposite direction (black dots = preferred direction (PD); circles = opposite direction (OD)). This difference persists after Gad2-activation (RP in control: PD 64 ± 7.7 %, OD 24 ± 6.4 %; p = 0.0001; RP Gad2-act.: PD 42 ± 6.6 %, OD 20 ± 6.2 %, p = 0.0126), but not L6-Ntsr1 activation (RP in control: PD: 55 ± 6.0 %, OD: 34 ± 6.9 %, p = 0.0022. RP L6-Ntsr1 act.: PD: 25 ± 7.0 %, OD: 21 ± 7.7 %, p = 0.3092). (*C*, *D*) A similar result was found for the excitatory L5 cells: RP in Ntsr1-cre mice in control: PD 52 ± 6.2 %, OD 36 ± 6.5 %, p = 0.0459; RP with L6-Ntsr1 activation: PD 37 ± 8.9 %, OD 30 ± 8.6 %, p = 0.6430. RP in Gad2-cre mice in control: PD 49 ± 7.8 %, OD 23 ± 7.2 %, p < 0.0001; RP with Gad2-activation: PD 39 ± 8.4 %, OD 18 ± 8.2 %, p = 0.0002). Two-way ANOVA matching both factors (Control/optogenetic activation and Preferred/Opposite direction) with reported p-values from Sidak´s multiple comparison test. The data thus indicate that after L6-Ntsr1 activation the cell is as likely to respond irrespective of the direction in which the whisker is deflected.

Supplementary Figure 7. Related to Figure 6.

Effect of L6-Ntsr1 activation on VPM thalamus

(*A*) Activation of L6-Ntsr1 cells alters the relay of high-frequency sensory stimuli (Mease et al., 2014). Here an example of a VPM neuron stimulated with four whisker deflections delivered at 4Hz (x-axis) with and without L6 photoactivation (40 sweeps). The response probability (number of whisker deflections evoking at least one spike / total number of whisker deflections) decreases in control but not with L6-Ntsr1 activation. *In vivo* single unit recording in VPM. (*B*) Raster plot illustrating the phenomenon seen in *A*. Each dot represents the time point of a spike (measured at the spike peak). Four whisker deflections at 4Hz (time: 300, 550, 800 and 1050 ms on the y-axis) displayed for 25 traces (x-axis). The response decreases with successive whisker deflections in control, but not with L6-Ntsr1 activation.

Supplementary Figure 8. Related to Figure 8.

L6-Ntsr1 cells can activate infragranular FS interneuron

Photoactivation of channelrhodopsin-expressing L6-Ntsr1 cells increased spiking in an infragranular fast-spiking (FS) interneuron (depth: 872µm, see Fig.1 and S1). (*A*) Control condition, no photoactivation. The response to a train of whisker deflections (4 whisker deflections at 4 Hz, every 5 s, repeated 160 times) showed facilitation (one-way ANOVA with repeated measure with post-hoc test between stimulation 1 and 4: p = 0.0462). (*B*) The same FS unit as in *A*. No obvious angular tuning (one-way ANOVA with repeated measures, p = 0.6418). Measurement were done in control condition, without photoactivation.

Figure S1

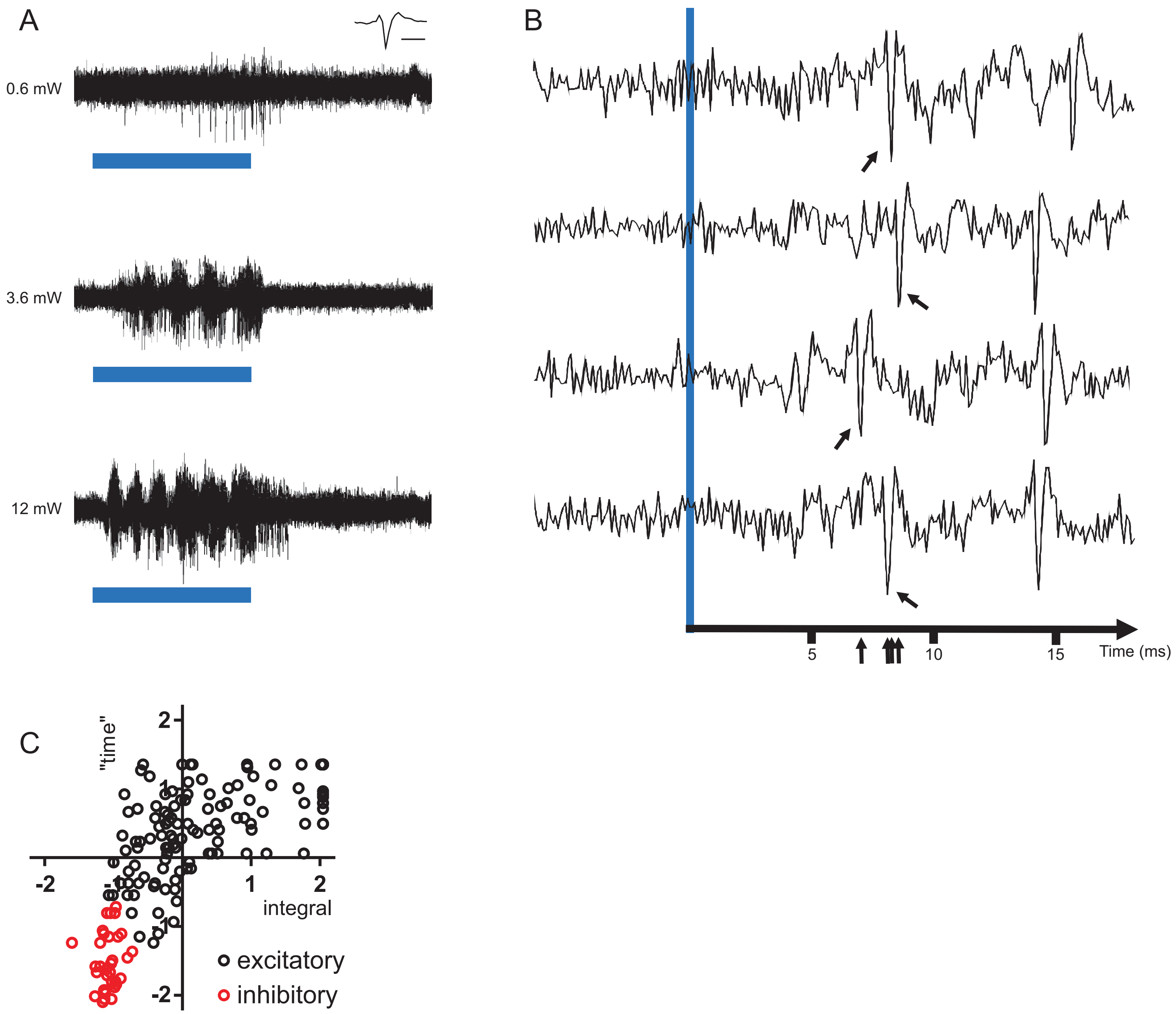
A
0.6 mW
3.6 mW
12 mW
B
5
10
15
Time (ms)
C
"time"
2
-2
1
2
integral
excitatory
inhibitory
-2

Figure S2

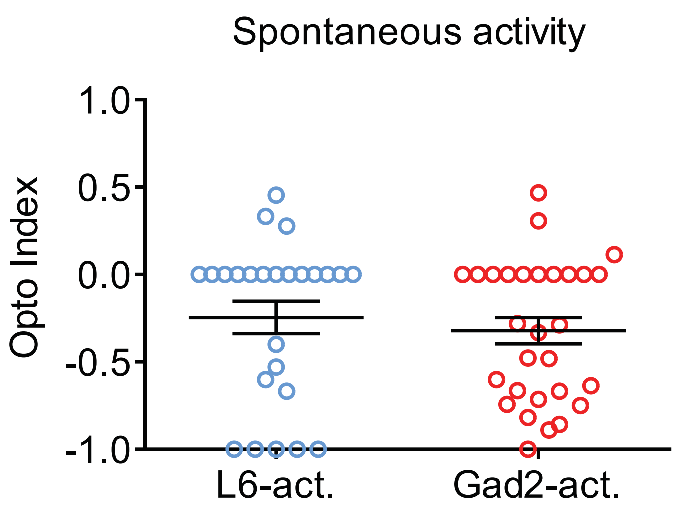

Spontaneous activity
1.0
0.5
0.0
-0.5
-1.0
Opto Index
L6-act.
Gad2-act.

Figure S3

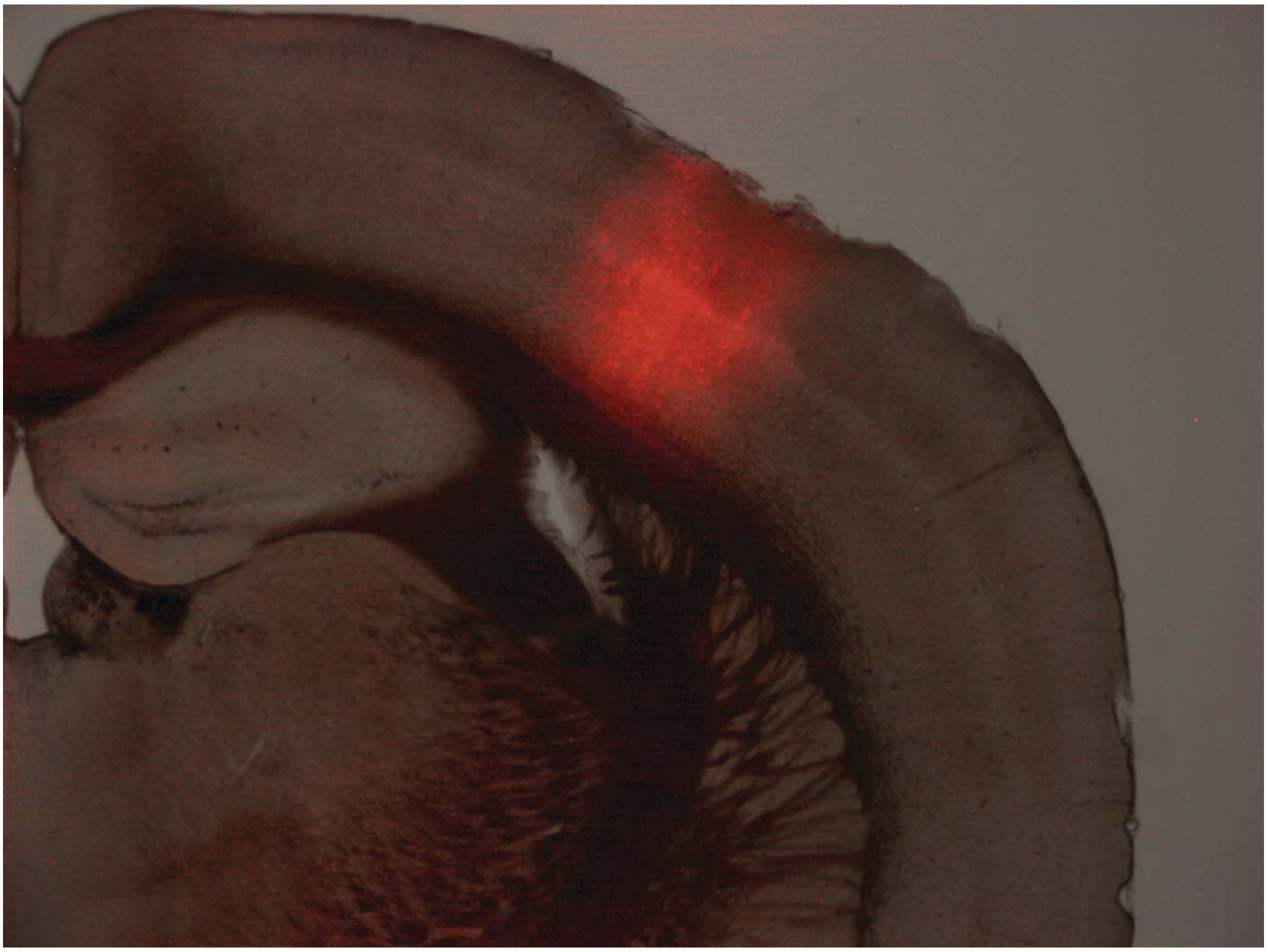

Figure S4

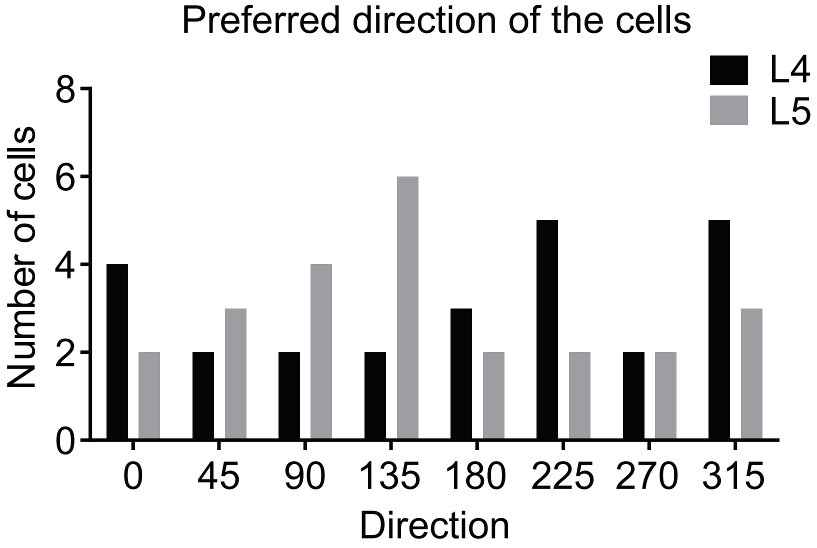

Preferred direction of the cells
L4
L5
Number of cells
8
6
4
2
0
0
45
90
135
180
225
270
315
Direction

Figure S5

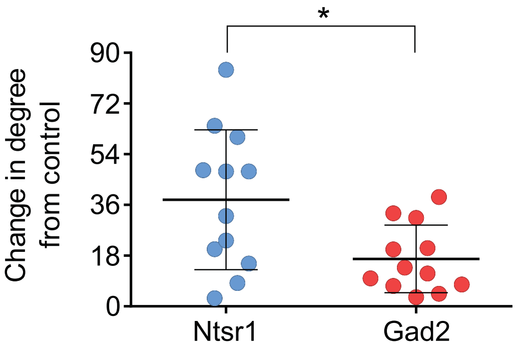

Figure S6

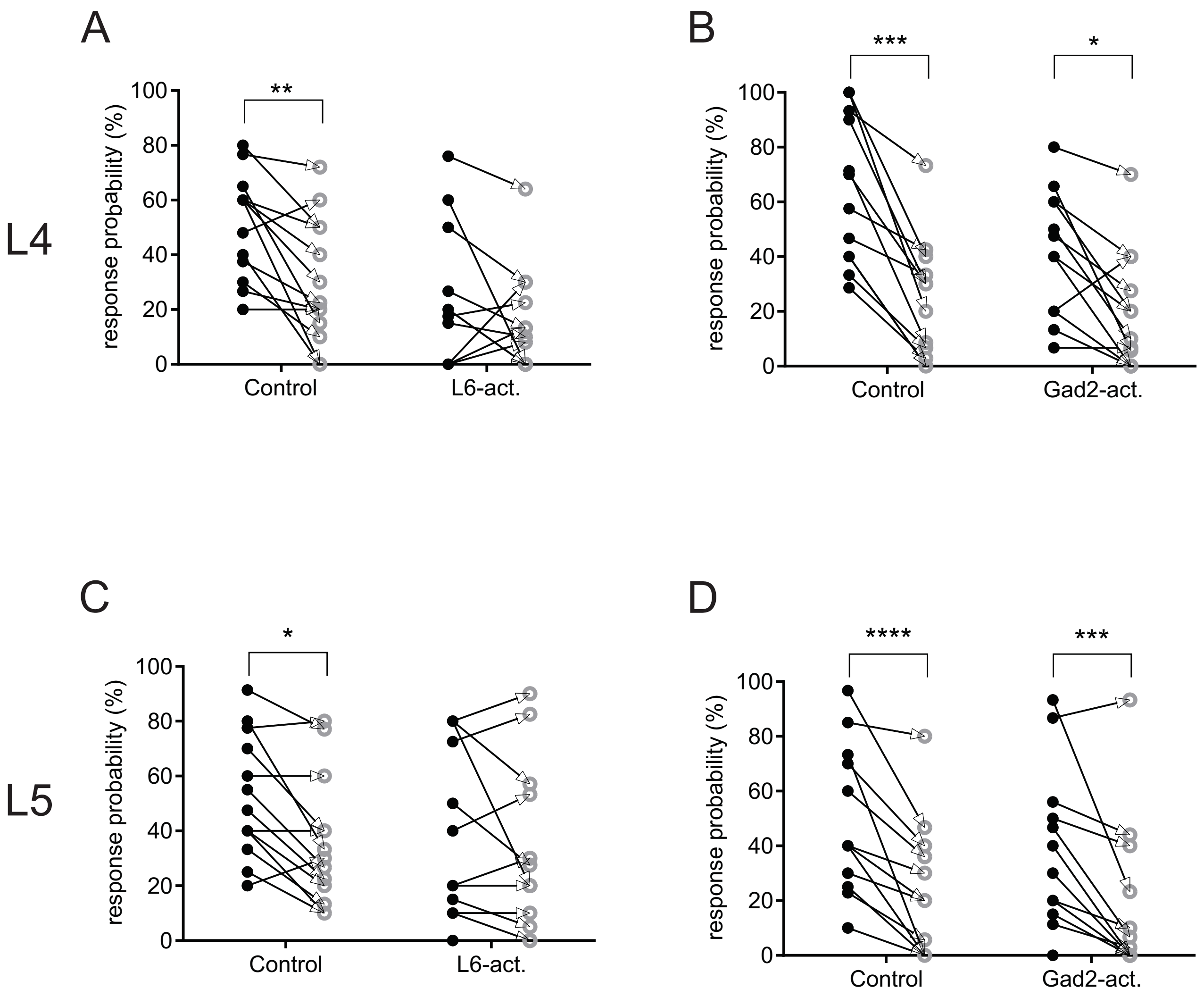

A
B
C
D
L4
L5
response probability (%)
100
80
60
40
20
0
**
***
*
****
Control
L6-act.
Gad2-act.

Figure S7

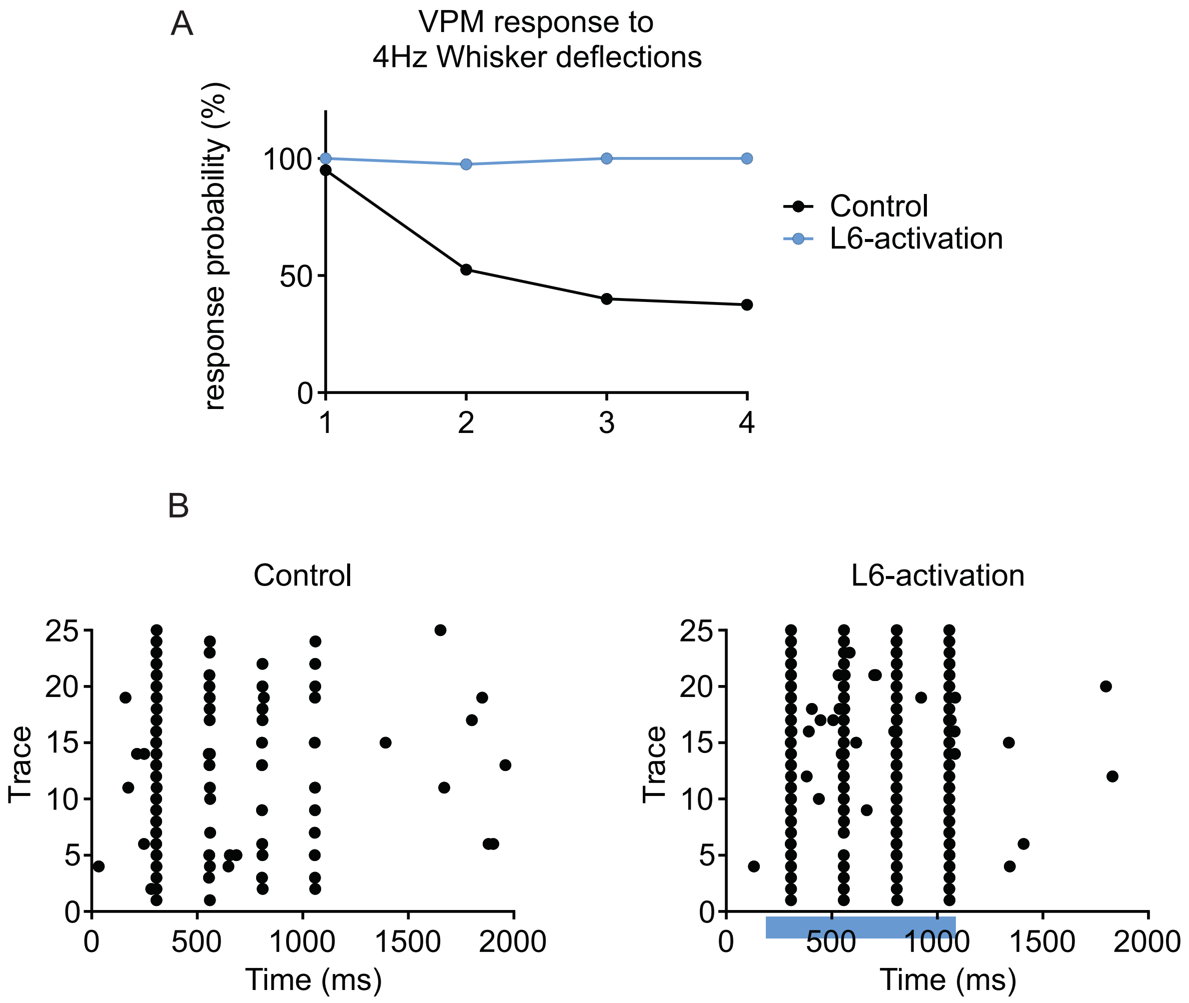
A
VPM response to
4Hz Whisker deflections
response probability (%)
100
50
0
1
2
3
4
Control
L6-activation
B
Control
L6-activation
Trace
25
20
15
10
5
0
0
500
1000
1500
2000
Time (ms)

Figure S8

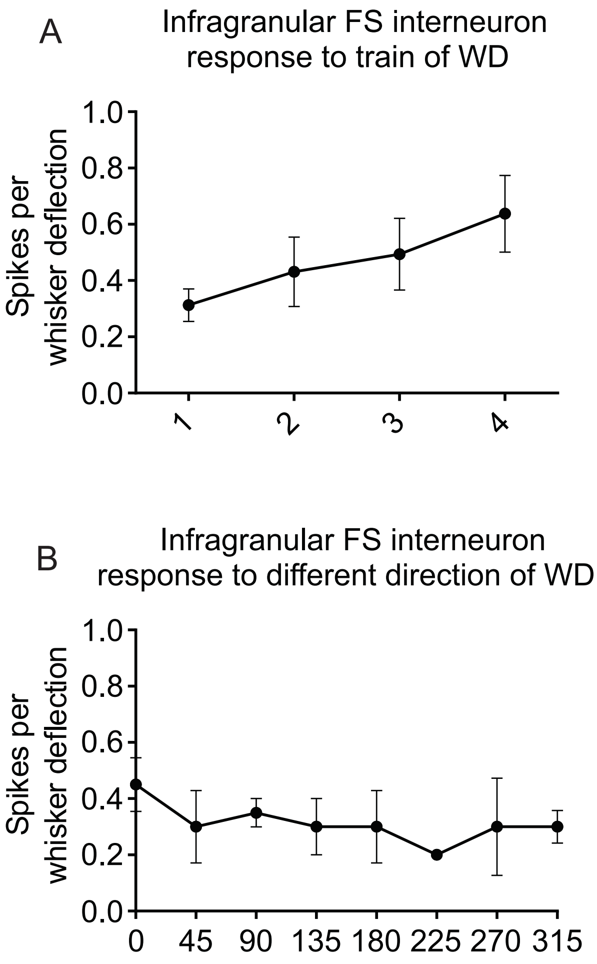
A
Infragranular FS interneuron
response to train of WD
Spikes per
whisker deflection
1.0
0.8
0.6
0.4
0.2
0.0
1
2
3
4
B
Infragranular FS interneuron
response to different direction of WD
Spikes per
whisker deflection
1.0
0.8
0.6
0.4
0.2
0.0
0
45
90
135
180
225
270
315
